# Prebiotic oligomerization and self-assembly of structurally diverse xenobiological monomers

**DOI:** 10.1038/s41598-020-74223-5

**Published:** 2020-10-16

**Authors:** Kuhan Chandru, Tony Z. Jia, Irena Mamajanov, Niraja Bapat, H. James Cleaves

**Affiliations:** 1 Space Science Center (ANGKASA), Institute of Climate Change, Level 3, Research Complex, National University of Malaysia, UKM, 43600 Bangi, Selangor Malaysia; 2 Department of Physical Chemistry, University of Chemistry and Technology, Prague, Technicka 5, 16628 Prague 6-Dejvice, Czech Republic; 3 Earth-Life Science Institute, Tokyo Institute of Technology, 2-12-1-IE-1 Ookayama, Meguro-ku, Tokyo, 152-8550 Japan; 4 Blue Marble Space Institute for Science, 1001 4th Ave, Suite 3201, Seattle, WA 98154 USA; 5 Indian Institute of Science Education and Research, Dr. Homi Bhabha Road, Pashan, Pune, Maharashtra 411 008 India; 6 Institute for Advanced Study, 1 Einstein Drive, Princeton, NJ 08540 USA

**Keywords:** Astrobiology, Polymer synthesis, Origin of life

## Abstract

Prebiotic chemists often study how modern biopolymers, e.g., peptides and nucleic acids, could have originated in the primitive environment, though most contemporary biomonomers don’t spontaneously oligomerize under mild conditions without activation or catalysis. However, life may not have originated using the same monomeric components that it does presently. There may be numerous non-biological (or “xenobiological”) monomer types that were prebiotically abundant and capable of facile oligomerization and self-assembly. Many modern biopolymers degrade abiotically preferentially via processes which produce thermodynamically stable ring structures, e.g. diketopiperazines in the case of proteins and 2′, 3′-cyclic nucleotide monophosphates in the case of RNA. This weakness is overcome in modern biological systems by kinetic control, but this need not have been the case for primitive systems. We explored here the oligomerization of a structurally diverse set of prebiotically plausible xenobiological monomers, which can hydrolytically interconvert between cyclic and acyclic forms, alone or in the presence of glycine under moderate temperature drying conditions. These monomers included various lactones, lactams and a thiolactone, which varied markedly in their stability, propensity to oligomerize and apparent modes of initiation, and the oligomeric products of some of these formed self-organized microscopic structures which may be relevant to protocell formation.

## Introduction

It is widely believed that the origins of life was the result of interactions among environmentally supplied organic compounds, which either self-organized or became organized via the input of environmentally supplied energy such as heat and light, which is the basis of the so-called heterotrophic hypothesis^1–3^. After some 60 years of study of prebiotic chemical synthesis, it is apparent that some organic compounds central to modern biochemistry may be derived from abiotic synthesis in varying yield under appropriate conditions^3–10^, but that such syntheses often also produce significant quantities of compounds not common in modern biochemistry^11–13^ as well as large amounts of as-of-yet uncharacterized material^14–16^.

Though non-covalent monomer assemblies may have been useful for the origins of life^17–19^, covalent polymers may also have been important, since regulation of defined-sequence polymer assembly is an important aspect of heritable information transfer. Biochemistry is largely mediated by weak bond-mediated interactions which precisely position functional groups supported by polymer scaffolds that maintain complex electron density topologies in time-persistent three-dimensional (3D) configurations. Specific 3D arrangement of electron density forms the basis of molecular recognition^20^, which is a prerequisite for biological catalysis^21^. If such configurations can be linked with feedback mechanisms for polymerization, a recursive interfacial molecular “language” can develop, and chemical systems capable of refining this language may become able to responsively evolve, entering the realm of Darwinian evolution.

To clarify the following discussion, while “polymers” are definitionally longer than “oligomers,” the cutoff between the two is somewhat gray. Here we use the prefix “poly-” to refer to specific processes, and use the prefix “oligo-” to describe general processes and products under 20 monomer units in length.

A considerable amount of previous research has examined biopolymer synthesis under “plausibly prebiotic conditions,” typically loosely understood to be chemistry allowed by crustal planetary temperature and pressure conditions under which water is a liquid, and resulting from concentrations of compounds robustly derivable from planetary atmospheric or geochemical synthesis or extraplanetary synthesis and delivery. Importantly, even under especially high rates of synthesis or delivery, concentrations of these compounds would likely have been low in many aqueous environments. Evaporation is frequently appealed to as a mechanism for concentrating organic compounds in primitive planetary environments^3,22^.

The generation of more complex organic compounds such as oligonucleotides and oligopeptides either by directly condensing monomers (e.g., nucleotides or amino acids) in solution^23,24^ or by using activating agents^25,26^ has been the focus of most research.

In the absence of activating agents, condensation under extreme conditions of heat or desiccation is often necessary when modern biomonomers are the reactants^26–30^. Activation chemistry is necessary to make long biooligomers partly because dehydration condensation is thermodynamically unfavorable in water. For example, peptide or phosphodiester bond formation in water at 25 °C entails a free energy change of about + 3–5 kcal mol^−1^^31^ and + 7.8–10.9 kcal mol^−1^^32^, respectively. To drive such reactions forward abiotically generally requires extreme temperatures that are often destructive to biomonomers^33,34^ and disruptive of the weak bonds that mediate the interactions which enable biopolymer folding^35^. The instability of biomonomers and biopolymers does not necessarily preclude the importance of high temperature syntheses for the origins of life, indeed all chemistry would be subjected to whatever selection is possible under any given environmental regime, but compounds that can be oligomerized under milder conditions might be preferable for these reasons.

Besides direct dehydration condensation, reversible ring opening polymerization (ROP) has been explored as a prebiotic method to produce covalent oligomers^36,37^. ROP is unusual in this context in that the addition of a ring monomer to a linear polymer does not release water as a product, and thus the dehydration must occur elsewhere in the overall reaction scheme, namely in the spontaneous interconversion of the ring and open chain forms of the monomer. The ring/open monomer equilibrium for five- and six-membered compounds compatible with ROP is generally too unfavorable for high molecular weight polymers to form^37^, but lactide and glycolide, six-membered dimers of lactic and glycolic acids, are exceptions, and they are major feedstocks for biodegradable plastic synthesis (e.g., ^38,39^). Oligomers or polymers formed from dehydration condensation of monomers which do not form thermodynamically stable small rings (such as peptides and nucleotides) may be able to form longer oligomers at low temperature equilibrium for this reason.

In other words, in order for dehydration condensation to be effective for any type of monomer (including ones which can engage in ROP), the monomer (which may technically be a dimer of amino acids in the case of a 2,5-diketopiperazine or a dimer of α-hydroxy acids (αHAs) in the case 2,5-diketo-1,4-dioxane) ring equilibrium must not be especially high so as to encourage tail-biting depolymerization. Other monomer types plausibly derived from prebiotic chemistry could thus help evolving chemical systems circumvent the above-mentioned thermodynamic bottleneck by removing the energetic requirement for condensation reactions while still enabling the formation of large catalytic and informational interfaces.

In the context of prebiotic chemistry, Orgel and co-workers were among the first to explore oligomerization of cyclic monomers, namely 2′,3′-adenosine monophosphate (cAMP), under drying conditions^40–42^. Since this study explored an oligomerization mechanism not used in contemporary biochemistry, it represents an example of the idea that there may have been “scaffolding” chemistries which helped to bootstrap the origins of life^43^. The idea of such scaffolding chemistries has been raised in other prebiotic contexts (e.g., the pre-RNA world^44,45^) among others^43^.

**Figure 1:**
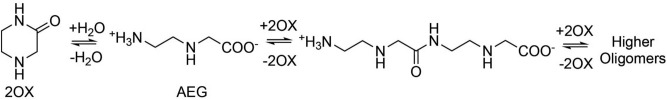
Reversible hydrolytic equilibrium between N-aminoethylglycine (AEG) and 2-oxopiperazine (2OX) in aqueous solution. AEG is shown in its zwitterionic form, which predominates near neutral pH. Subsequent reaction between ring-opened and ring-closed, or directly between ring-opened forms may give rise to oligomers.

Modern biological compounds have typically been explored as principle targets of prebiotic synthesis, but several efforts have focused on non-biological but equally plausible prebiotic molecules. For example, Miller and coworkers^46^ showed that the components of peptide nucleic acid (PNA)^47^ (a non-biological polymer), including the N-acetic acid modified bases and the backbone monomer N-aminoethylglycine (AEG), can be produced using plausible prebiotic precursors at extreme dilution. They also showed that AEG undergoes ring-closing dehydration to give an equilibrium mixture with 2-oxopiperazine (2OX) (Fig. 1)^48^.

Importantly, the AEG/2OX equilibrium is significantly different from that of the analogous glycylglycine/diketopiperazine (DKP) equilibrium resulting from α-amino acid condensation. The important difference between these two examples is the propensity for ring-closure of the monomers. Simply put, modern biological monomers may be difficult to oligomerize due to their propensity to form thermodynamically stable rings which may halt elongation. At the same time, there may be other non-biological compounds which are not able to generate the desired properties of modern plastics, but which may make longer oligomers than modern biomonomers are able to.

2OX (a closed ring monomer), when sufficiently concentrated, is able to react with AEG (open ring monomer) to give an AEG dimer, which is in equilibrium with AEG and 2OX, and this dimer is further able to react with a second molecule of 2OX to give an AEG trimer, again in equilibrium, and so on.

This type of oligomerization chemistry produces a dynamic equilibrium polymerization^49,50^ which can yield oligomers of considerable complexity if multiple monomer types are involved. For example, the chemistry shown in Fig. 1 could also be accomplished using a mixture of AEG, DL-N-aminoethylalanine, DL-N-aminoethylaspartate, etc. which are likely to be as prebiotically plausible as AEG, being derivable from the same Strecker-like synthetic pathways.

Many other small plausibly prebiotic monomer types may also lend themselves to this kind of combinatorial diversification. For example, we recently demonstrated the facile generation of prebiotically plausible dynamic combinatorial polyester libraries from mixtures of α-hydroxy acids (αHAs) with varied side chains^51^. The synthesis of commercial polylactide occurs principally via a ROP process, thus both poly-AEG and poly-αHA are examples of simple abiotic polymers that *can* form via various simple dehydration mechanisms.

In the present study, we examined a diverse suite of unexplored plausibly prebiotic monomers that can test whether ring-closure equilibria are limiting for oligomerization under mild wet-drying conditions. Initiation and co-oligomerization with compounds such as the likely prebiotically abundant amino acid glycine (Gly) is robust, which suggests that a variety of non-biological homo- and heteropolymers composed of various monomer types could have been present in prebiotic environments, helping sculpt the prebiotic catalytic landscape. Some of these systems also spontaneously form non-covalent micron-scale structures of possible relevance for the formation of compartments, perhaps leading to non-biomolecular-based protocells.

## Results and discussion

### Cyclic monomer oligomerization

**Figure 2:**
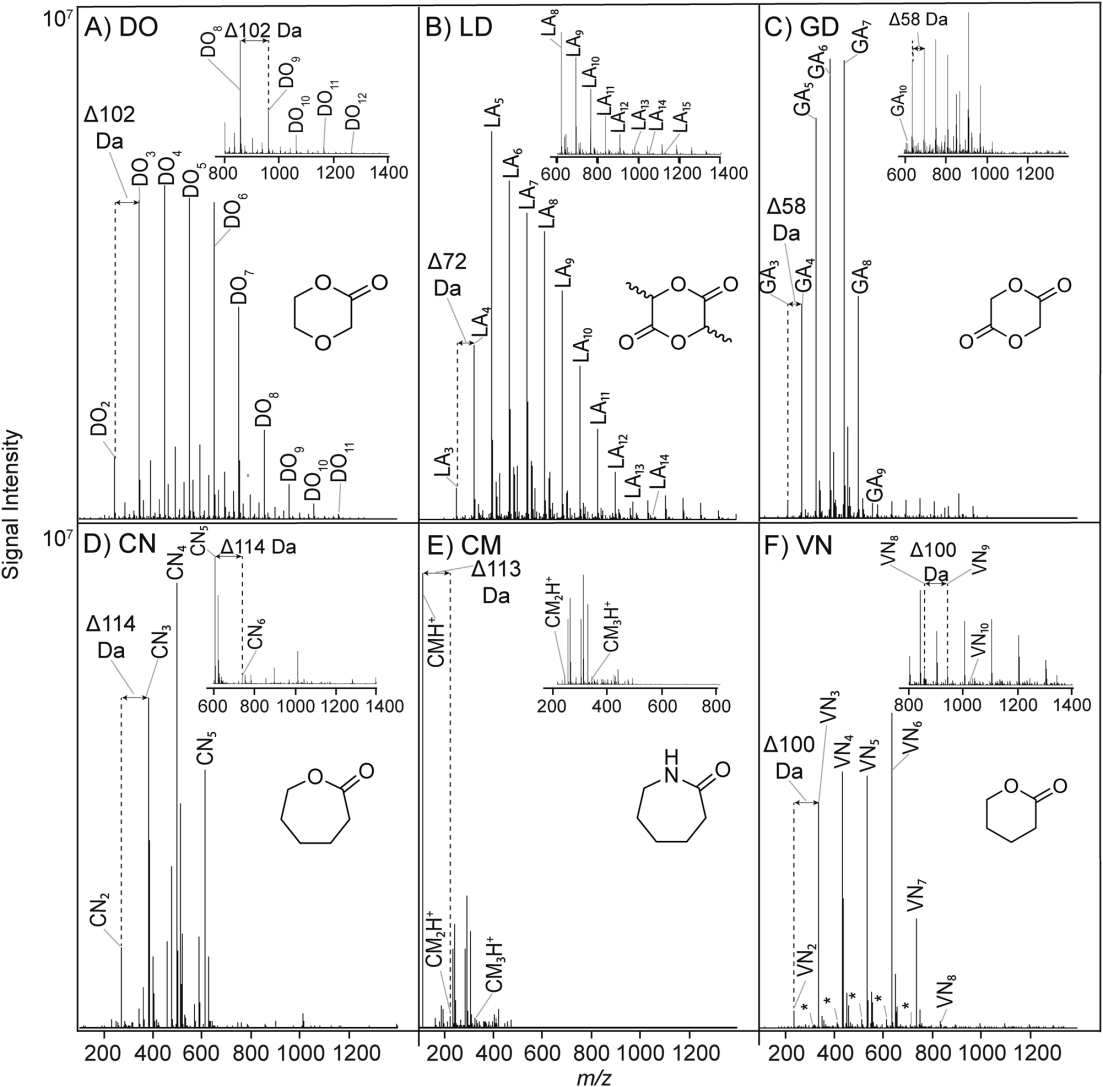

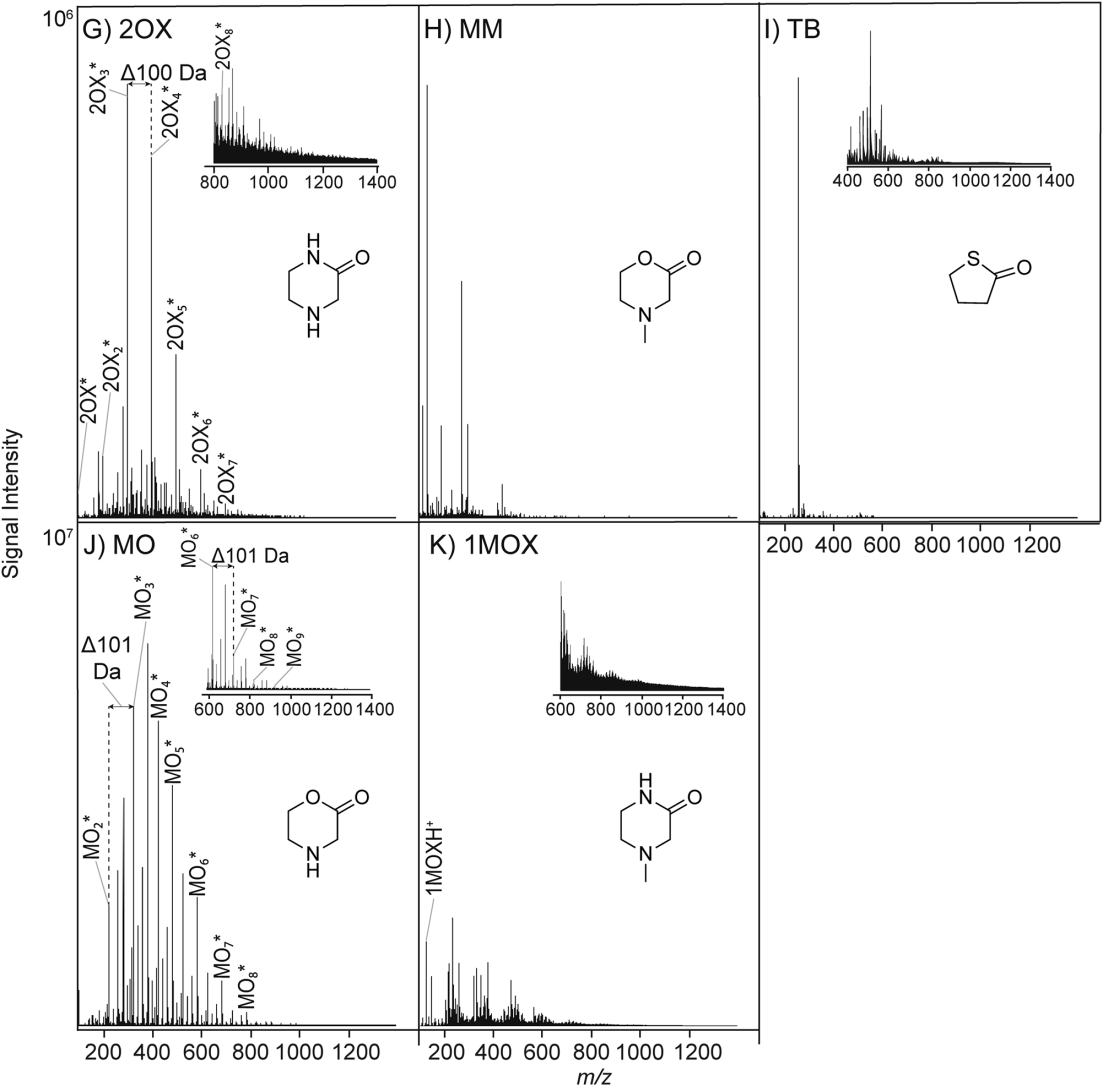
Positive mode Electrospray Ionization Quadrupole Time-of-Flight Mass Spectrometry (ESI-QToF-MS) mass spectra of oligomers obtained from drying monomer solutions over 24 h at 60 °C. (**A**) 1,4-dioxan-2-one (DO), (**B**) lactide (LD), (**C**) glycolide (GD), (**D**) ε-caprolactone (CN), (**E**) ε-caprolactam (CM) (**F**) δ-valerolactone (VN), (**G**) 2-oxopiperazine (2OX) (**H**) 4-methylmorpholin-2-one (MM), (**I**) γ-thiobutyrolactone (TB), (**J**) morpholine-2-one (MO) and (**K**) 1-methyl-3-oxopiperazine (1MOX). Negative mode spectra for 60 °C and positive and negative mode spectra for 80 °C and 100 °C experiments are provided in Figures SI1 and SI2. The repeating unit for GD and LD is shown as glycolic acid (GA) and lactic acid (LA) due to the hydrolysis of GD and LD to their respective GA and LA units (see text for further explanation). Insets for higher mass ranges are not shown for CM, MM and 1MOX due to their low intensities. For clarity, only MNa^+^ adduct (M = mass) peaks are labeled unless stated otherwise, except for 2OX and CM for which MH-H_2_O^+^ adducts and MH^+^ adducts, respectively, are prominent in the spectra. Asterisks highlight masses suggesting water loss.

A variety of compounds which could be expected to undergo reversible ring-opening in water were explored. We thus examined the simple drying reactions of 1,4-dioxan-2-one (DO), lactide (LD), glycolide (GD), ε-caprolactone (CN), ε-caprolactam (CM), δ-valerolactone (VN), 2-oxopiperazine (2OX), 4-methylmorpholin-2-one (MM), γ-thiobutyrolactone (TB), morpholine-2-one (MO) and 1-methyl-3-oxopiperazine (1MOX). This selection was meant to sample a variety of ring sizes and structural motifs (structures are shown in Fig. 2) and was in general restricted to structures with plausible prebiotic syntheses (see below).

Many of these monomers oligomerized significantly at the studied temperatures (60–100 °C) when dried from unbuffered solution. The most intense peaks detected by direct infusion electrospray ionization mass spectrometry (ESI–MS) in positive ionization mode were generally assignable as the sodiated parent ion (MNa^+^) adducts, though other types of adducts (e.g., MH^+^) were also observed (Figs. 2 and SI1). In negative ionization mode, the most intense peaks were generally assignable as MH^-^ species (Fig. SI2). In Fig. 2, results are shown with normalized signal intensity, which may be a reasonable proxy for product yield. We discuss the results here according to shared chemical features.

**Figure 3:**
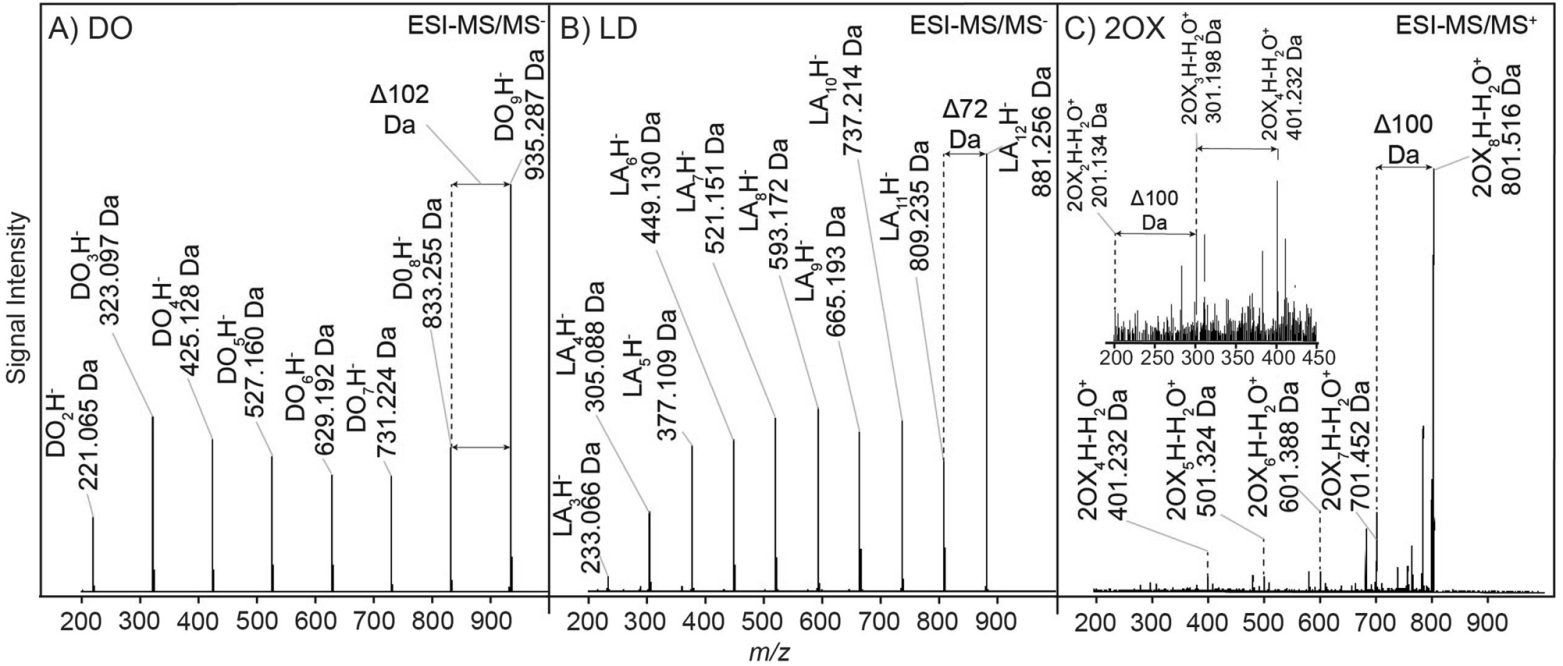
Representative ESI-QToF-MS/MS fragmentation spectra of selected ROP oligomers formed at 80 °C. (**A**) DO_9_ (MH^−^, 935.287 Da, negative ionization mode) formed from DO, (**B**) LA_12_ (MH^-^, 881.256 Da, negative ionization mode) formed from LD and (**C**) 2OX_8_ (MH-H_2_O^+^, 801.516 Da, positive ionization mode).

These results reveal several important phenomena. Some monomers oligomerize in predictable fashions, while others do not. Dioxanone (DO) (Fig. 2A) oligomerized measurably, and DO oligomers were detected at all temperatures (60 °C, 80 °C and 100 °C) studied. A Δ102.032 Da mass increment corresponding to a repeating unit of –(O(CH_2_)_2_OCH_2_C(=O))– was evident. Oligomers up to 12-mers were easily recognizable using both positive and negative mode MS (Figs. SI1A and SI2A). Oligomers with masses indicating water loss (− 18.011 Da), which may be cyclic or otherwise dehydrated, were also detected at all temperatures in negative mode (Fig. SI2A), indicating that more than one type of oligomer is generated. MS/MS analysis provided clear assignable sequential monomer loss from a fragmented DO_9_H^+^ ion (see Fig. 3A), though other length oligomers gave similar fragmentation spectra.

LD and GD are the cyclic dimers of lactic acid (LA) and glycolic acid (GA) respectively. Figure 2B,C suggest that significant hydrolysis of LD and GD and/or oligomer chain-swapping occurs during oligomerization under drying conditions, since both LD and GD produce both odd and even length oligomers of similar relative intensity. LD sample spectra showed a repeating LA unit of Δ72.021 Da (Fig. 2B) corresponding to a repeating –(OCH(CH_3_)C(=O))– unit. Monodehydrated oligomers of LA up to 13-mers were detected between 60 and 100 °C, and 15-mers were detected at 80 °C. Additional mass ladder series with the Δ72.021 Da increment were visible well beyond m/z 1000. MS/MS analysis (Fig. 3B) of an isolated LA_12_ peak clearly showed the expected lactic acid residue loss. GD behaved similarly to LD, with both odd and even length oligomers detectable with a mass increment of GA units of Δ58.005 Da (Fig. 2C), corresponding to a repeating –(OCH_2_C(=O))– unit. Monosodiated GA oligomers up to 9-mers were detected at all temperatures, and 11-mers were detected at 80° C in positive ionization mode. Other unknown adduct type mass ladders showing the Δ58.005 Da increment well beyond m/z 600 were evident (Figs. SI1C and SI2C).

The seven-membered ring monomers CN and CM also oligomerized by drying between 60–100 °C and showed a Δ114.068 Da mass ladder corresponding to a –(O(CH_2_)_5_C(=O))-unit or a Δ113.084 Da mass ladder corresponding to a –(NH(CH_2_)_5_C(=O))–unit, respectively. (Fig. 2D,E). CN Oligomers up to 6-mers were detected at 60 °C in both positive and negative ionization mode (Figs. 2D, SI1D and SI2D) alongside unassigned adduct series which also displayed the repeating monomer mass of Δ114.068 Da. CM oligomers up to 3-mers were detected in positive ionization mode at all temperatures studied (Figs. 2E and SI1E) and in negative ionization mode only at 60 °C (Fig. SI2E).

For VN (Fig. 2F), MNa^+^ oligomer adducts up to 7-mers were detected at all temperatures studied (60–100 °C), and oligomers up to 10-mers were detected at 60 °C. The VN oligomers showed a mass increment of Δ100.052 Da corresponding to a –(O(CH_2_)_4_C(=O))– unit. VN also gave peak series corresponding to oligomers with a single additional water loss which are likely either cyclic or terminally dehydrated (Figs. 2F, SI1F and SI2F).

Reactions of 2OX at 60 °C (Fig. 2G) and 80 °C (Fig. SI1G) gave assignable MH–H_2_O^+^ oligomer peaks in positive ionization mode that may similarly correspond to cyclic or otherwise dehydrated oligomers. No assignable oligomer peaks were detected from reactions conducted at 100 °C, and the baseline became increasingly complex with increasing reaction temperature, likely due to monomer degradation during heating. Linear 2OX oligomers (or AEG oligomers) up to 7-mers were detectable at 60 °C only in negative ionization mode (Fig. SI2G).

Figure 3 shows MS/MS analyses of selected samples shown in Fig. 2, which allows analysis of the oligomer composition, e.g., the extent to which each is composed of many possible polymer sequence permutations. For 2OX, both negative and positive mode spectra showed a mass increment of Δ100.064 Da corresponding to the repeating –(NH(CH_2_)_2_NHCH_2_C(=O))– unit. MS/MS fragmentation of an isolated 2OX 8-mer (Fig. 3C) also showed the expected ~ 100 Da monomer loss.

MM (Figs. 2H, SI1H and SI2H) and TB (Figs. 2I, SI1I and SI2I) did not show evidence of efficient oligomerization in positive or negative ionization mode at any temperature.

MO (Fig. 2J) showed the expected mass increment of Δ101.048 Da corresponding to a repeating –(O(CH_2_)_2_N(H)CH_2_C(=O))– monomer unit. Oligomers up to dehydrated and/or cyclic 8-mers were detected in positive ionization mode (Fig. SI1J). These were only evident from reactions conducted at 60 °C, while in negative mode, other unassigned adduct series were detected (Fig. SI2J).

1MOX gave a complex spectrum with little evidence for direct oligomerization but with a mass “cluster” with a period approximately that of the monomer mass (~ 114 Da, Fig. 2K), suggestive of monomer decomposition and the occurrence of significant competing side reactions (see below for discussion). This apparent decomposition was evident even at 60 °C (Fig. 2K) and became more pronounced at higher temperatures (Figs. SI1K and SI2K). The importance of this generation of increasingly complex spectra as a function of increasing temperature, especially from rings containing one or more nitrogen atoms is discussed in further detail below.

**Figure 4:**
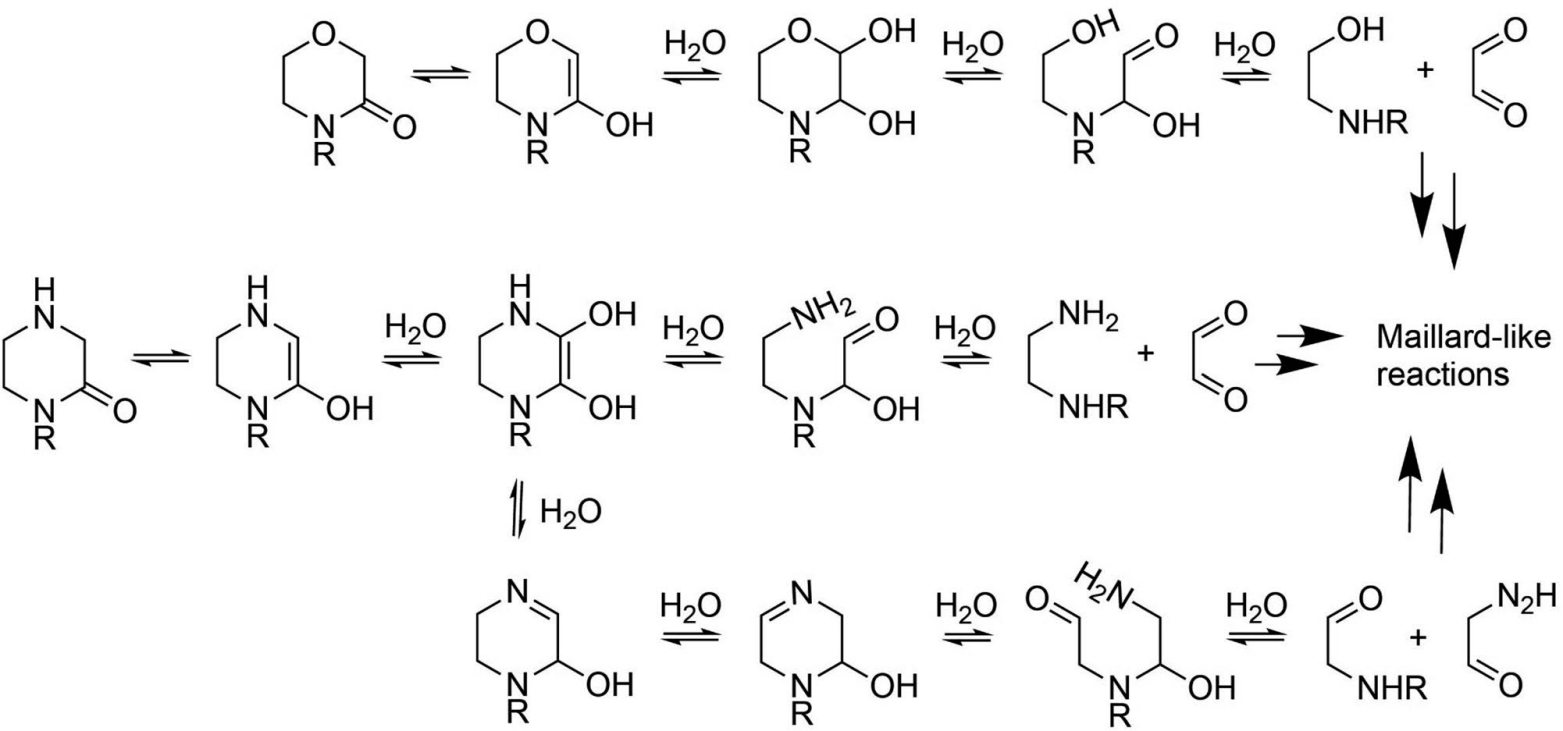
Proposed mechanisms for the degradation of MO and 2OX monomer derivatives to give complex tarry mixtures.

Monomers containing at least one ring nitrogen (e.g., CM, 2OX, MM, MO and 1MOX, see Fig. 2E,G,H,J,K) oligomerized poorly by themselves, and showed evidence of degradation under the reaction conditions studied, as noted both by the deep browning of the residues in the test tubes after heating and the complex mass spectra of the products (e.g., see Figs. 2 and SI3). This can reasonably be explained by the degradation of the starting materials to give reactive amino and aldehyde species which undergo reactions similar to those occurring during the Maillard process (e.g.,^52^). The reaction of glyoxal, one of the possible products of this degradation, and cyanamide has been studied in prebiotic contexts and found to produce complex mixtures^53^. A plausible set of mechanisms for the degradation and spectra observed here is presented in Fig. 4.

These reactions likely involve the complex interplay of solution phase and dry phase mechanisms^22^, which may be pH, concentration and temperature dependent. To explore this further, the chemistry of 2OX as a model compound in solution at various concentrations (2–200 mM) was studied by ^1^H NMR (Fig. SI4). At 25 °C between pH 5 and 7, the ring form is favored, and no ring-opening was observed in solution after 6 months of incubation at 25 °C. However, at pH 9, ring hydrolysis was measurable over the course of a few hours, and at this pH the equilibrium (AEG/2OX) appeared to be ~ 1, which is consistent with previous results^48^. More concentrated solutions gave evidence of the formation of oligomers even in solution, as suggested by DOSY spectroscopy (Fig. SI4F), however these results remain to be confirmed by mass spectrometry. Thus, in some cases oligomerization may be able to occur spontaneously at room temperature in concentrated solution.

### Co-oligomerization of cyclic monomers with glycine

We further examined the reaction of this set of compounds in the presence of Gly, which we reasoned could serve as an oligomerization initiator leading to both co-oligomers with a single initiator residue at one end and more heterogeneous co-oligomers depending on the dominant reaction mechanism (ROP or simple condensation). The addition of Gly to drying solutions of these monomers produced a variety of mixed oligomers, and many of the cyclic monomers oligomerized readily under the conditions explored here, either incorporating Gly or showing enhanced oligomerization in the presence of Gly even without its incorporation in the resulting oligomers.

**Figure 5:**
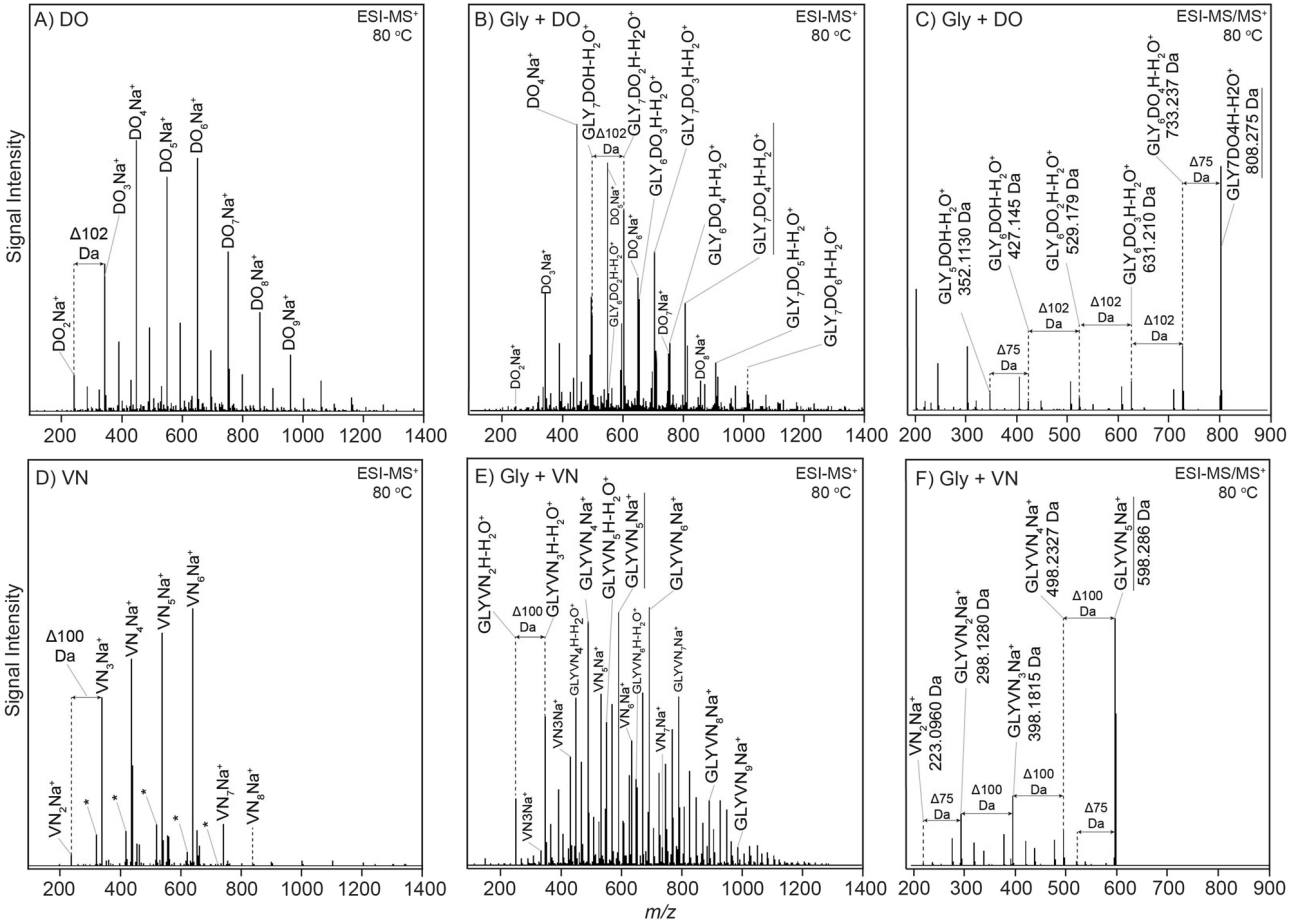
ESI-QToF-MS and MS/MS analyses of selected oligomeric products with or without Gly. DO in the (**A**) absence or (**B**) presence of Gly at 80 °C. (**C**) MS/MS analysis of the 808.275 Da (GLY_7_DO_4_H-H2O^+^) parent peak underlined in **B**. Oligomeric products of VN in the (**D**)absence or (**E**) presence of Gly at 80 °C, and (**F**) MS/MS analysis of the 598.286 Da parent peak (GLYVN_5_Na^+^) underlined in **E**.

As examples of this, Fig. 5 shows mass spectra of the products of reactions of DO or VN with Gly. The effects of the presence of Gly are readily apparent (see Fig. SI5A,F for further results from this series), and it is apparent that such reactions can be quite nuanced in their behavior.

DO presented especially sparse and easily interpretable spectra. When DO was dried with Gly, DO homo-oligomers and mixed Gly + DO hetero-oligomers were formed at all temperatures studied (60–100 °C, see Fig. SI5A). Oligomers up to 13-mers were formed at all temperatures. The extent of oligomerization increased with increasing temperature and mixed cyclic or allyl alcohol-terminated oligomers showing one additional water loss (MH-H_2_O^+^), were formed at 80 °C and above.

MS analysis of Gly/DO reactions conducted at 80 °C showed new peaks relative to reactions lacking Gly corresponding to mixed Gly-DO oligomers (Fig. 5A,B). MS/MS analysis of selected prominent peaks from these reactions suggested each was composed of many possible polymer sequence permutations, with their identification limited only by the resolution of the MS analysis. For example, the isolated peak identified as Gly_7_DO_4_H-H_2_O^+^ (Fig. 5C), assuming it is a linear dehydrated oligomer (it could also be cyclic, or a mixture of both types), could have 120 (10!/(7!3!)) permutation sequences that could contribute to the isolated peak’s intensity.

Similarly, in contrast to the VN-only spectra (Fig. 5D), when Gly was dried with VN (Fig. 5E), a variety of linear and cyclic Gly/VN heterooligomer peaks appeared in the spectra. With increasing reaction temperature, the spectra became more complex than can easily be explained by co-oligomerization-generated product diversity (Fig. SI5F). Mixed linear oligomers up to 10-mers were detected in the 80 °C samples, and monodehydrated or cyclic oligomers were detected at all temperatures studied (Fig. SI5F). MS/MS of a high intensity selected peak identified as GlyVN_5_Na^+^ (Fig. 5F) showed evidence of positional variance of a single Gly residue that could have five permutation sequences, assuming VN ring-opening is initiated by Gly.

Co-oligomerization of Gly with CN (Fig. SI5D), also produced mixed oligomers at all temperatures, and Gly with MO produced mixed oligomers at 60 °C only (Fig. SI5J).

Gly may not only be an initiator that can incorporate into various oligomer systems, but it may also serve as a catalyst promoting oligomerization. This is exemplified by the 2OX system: reaction of 2OX without Gly (Fig. SI1G) did not show oligomerization at temperatures above 80 °C, but when Gly was added, oligomerization up to 5-mers did occur at 80 °C (Fig. SI5G), but these oligomers did not incorporate Gly.

In some cases, Gly appeared to act both as an initiator *and* as a catalyst. MM reacted alone did not oligomerize at any temperature studied (60–100 °C, Figs. SI1H and SI2H), but when Gly was reacted with MM (Fig. SI5H), MM oligomers up to 5-mers were detected (for which Gly presumably acts as a catalyst), as well as an MM oligomer series containing a single Gly residue (e.g., GlyMM_3_H^+^) (for which Gly could serve as an initiator). However, no oligomerization was observed with CM, TB or 1MOX in the presence of Gly (Figs. SI5E,I,K).

The cyclic dimer ɑHAs (LD and GD) display especially complex behavior in the presence of Gly, thus they are discussed in greater detail here. The average oligomer length was somewhat longer in the absence of Gly, and thus Gly appears to limit the chain length that can be reached.

For the GD/Gly system, closely clustered mass peaks were evident which are explainable as the ~ 1 Da difference between Gly and GA monomers (Fig. SI5C). The products likely include various mixed amide/ester oligomers, e.g., depsipeptides, of large sequence diversity. This is not surprising, and depsipeptide formation from Gly and GA has been explored extensively previously (e.g.,^54^). There are 2^n^ (where n = the polymer length) possible linear sequences and n + 1 unique masses for any two-monomer system for which each monomer has a unique mass (e.g., for n = 2, there are three unique mass species, two heterobaric homodimers (e.g., GAGA and GlyGly) and two isobaric heterodimers (e.g., GAGly and GlyGA). A 7-mer (n = 7) mixed sequence oligomer derived from two monomer types may have any one of eight unique masses, and a mass peak may represent up to 35 (e.g., 7!/(4!*3!), for the most diverse set containing three of one monomer type and four of the other) unique, though isobaric, sequences.

**Figure 6:**
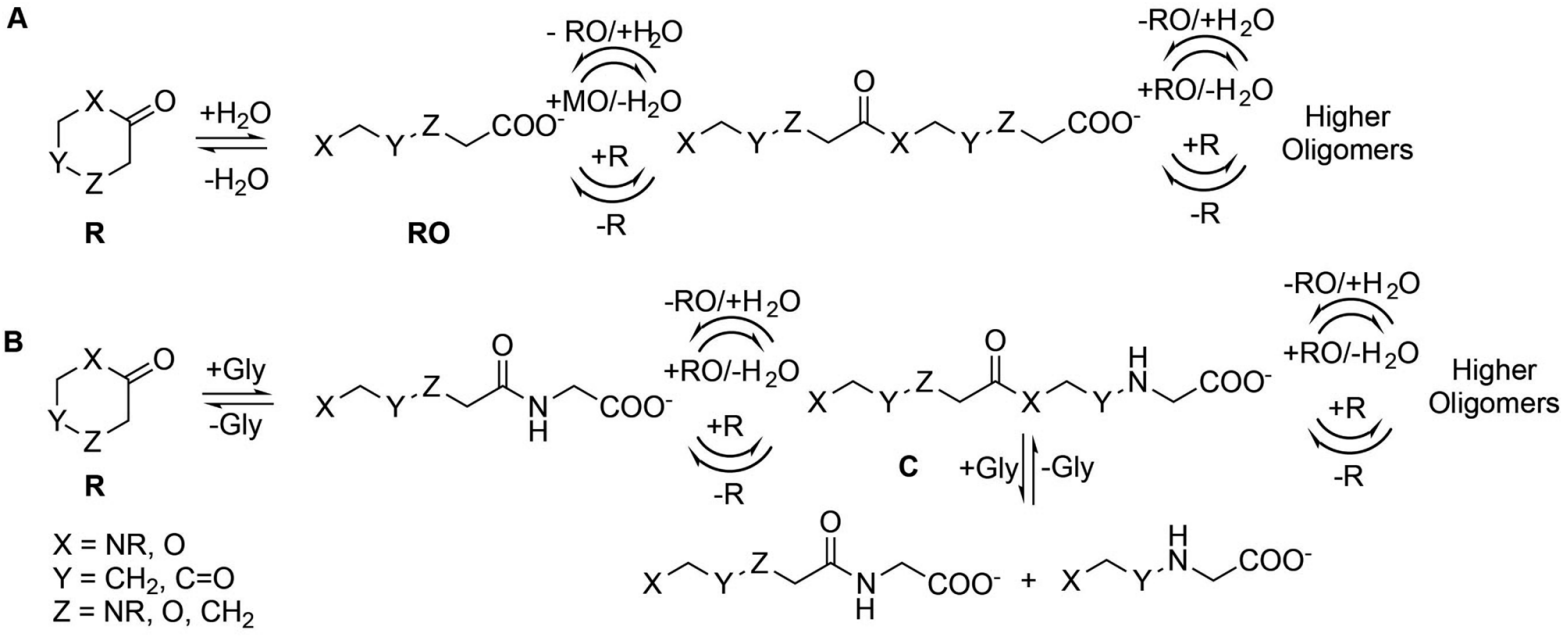
Mixed initiation, elongation and diversification mechanisms for heterogeneous oligomers derived from drying reactions of the cyclic monomers studied in this work. *R* ring-closed monomer, *RO* ring-opened monomer. Proposed mechanisms include (**A**). hydrolytic monomer ring opening followed by direct condensation and/or ring-opening oligomerization, (**B**) Ring opening initiated by glycine, followed by oligomer extension via direct condensation and/or ring-opening oligomerization, and (**C**) internal scission of oligomers by Gly and amide exchange. These three mechanisms can collectively account for much of the diversity of product ESI–MS and ESI–MS/MS masses observed here, but other mechanisms may also be operative.

A diagram explaining the combination of oligomerization reaction mechanisms likely contributing to the observed products is shown in Fig. 6.

There was also a pronounced effect of temperature on the length and sequence diversity of the products obtained when LD or GD were heated with Gly (Fig. SI5B,C). These systems gave co-oligomers that generally contained more LA or GA than Gly residues at all temperatures examined. This can be seen clearly in the spectra obtained from reactions conducted at 60 °C (Figs. SI1B and SI5B; Figs. SI1C and SI5C), where only one Gly residue was detected in the highest intensity mass peaks. Even at higher temperatures, although an increase in Gly content in the product co-oligomers relative to lower temperature reactions was apparent, the total LA or GA content was still greater than the Gly content, though depsipeptide oligomers containing up to 5 Gly residues were measurable (e.g., Gly_5_GA_8_H^+^) at 100 °C (Fig. SI5C). The longest co-oligomer identified at 100 °C was the 20-mer Gly_3_GA_17_Na^+^ (Fig. SI5C). This is concordant with previous results^54^, and thermodynamics^31,55^.

Glycine homo-oligomers were also formed within the CN and GD systems at 80 °C and 100 °C (Fig. SI5C,D), where up to pentamers of oligogycine were detected at 100 °C for the CN + Gly mixed system (Fig. SI5D). The formation of glycine oligomers, especially in higher temperature mixed monomer systems, is most likely due to the increase in the equilibrium constant for peptide formation^31^. Nonetheless, although oligoglycine was only prominent in these two systems, there is reason to believe that the formation of short oligopeptides may have also occurred in all other mixed systems, albeit showing less intense mass spectral peaks.

### Emergent higher order structure formation

**Figure 7:**
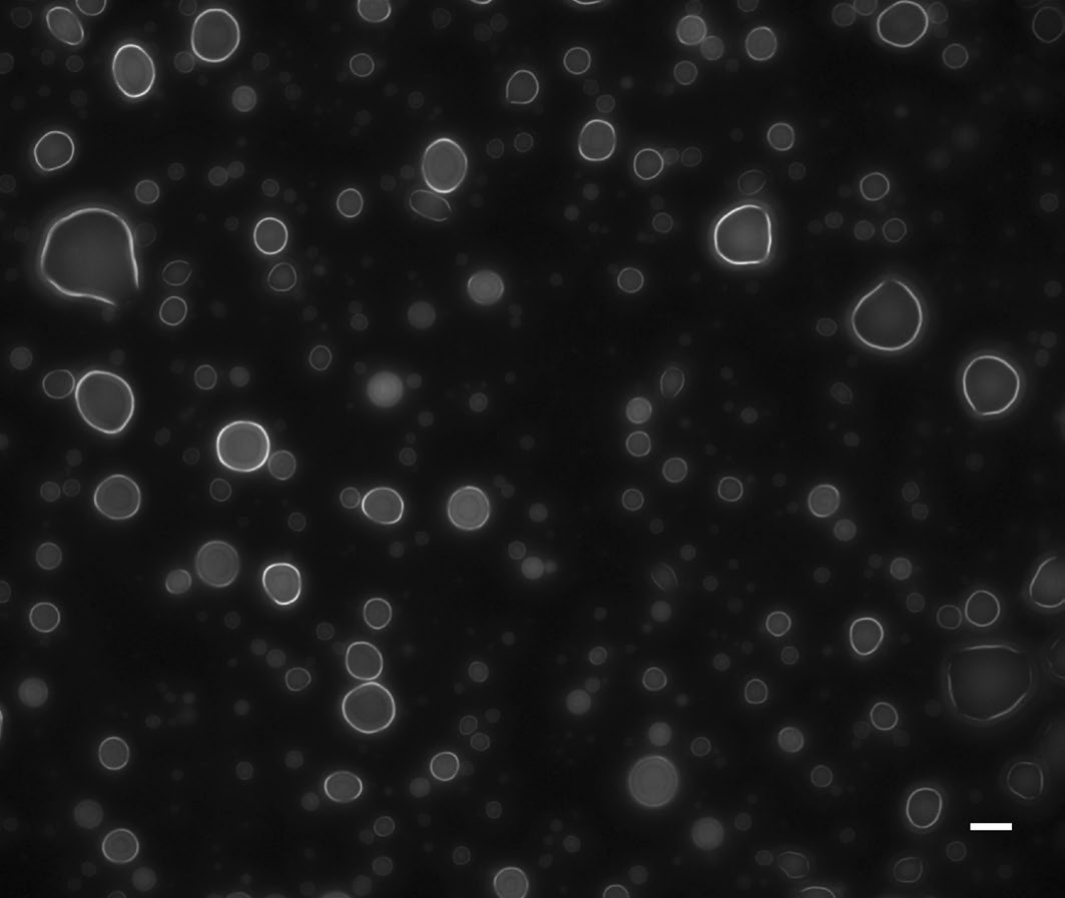
Micrograph of the structures obtained after rehydration of a drying reaction containing GD and Gly at 80 °C. Scale bar is 10 µm.

The abiotic synthesis of chemistries which may have led to assembly of proto cellular structures capable of encapsulation remains an important area in prebiotic chemistry research^56,57^. Observation of the reaction systems explored here using light microscopy revealed multiple types of emergent micron-scale aggregates and assemblies. This is somewhat surprising as many of these oligomers do not appear at first glance to have obvious structural attributes that would cause such behaviors. We have examined this phenomenon and its scope in greater detail and the results are reported elsewhere^58^, and we note here only that these structures range in nature from globules to rod-like aggregates, depending on the composition. A micrograph of the products of a Gly/GD reaction are shown in Fig. 7 (further particle analysis is shown in Fig. SI6). The products of these reactions, which include various heterogeneous short mixed peptides and oligoesters which likely have the capacity to hydrogen bond among each other, spontaneously form spheroidal globules (simultaneously with other rod-like aggregates, see Fig. SI7) which suggests they develop some degree of surface tension.

### Prebiotic relevance

The development of autocatalytic and self-reproducing chemical systems is a central problem in understanding the origins of life^59,60^. These systems could in principle be based on many polymer types^43–46,61^, and which types were prevalent in prebiological environments would depend on the balance of monomer synthesis, i.e., abundance as a result of robustness of prebiotic synthesis under a given set of conditions, and reactivity, including robustness of elongation mechanisms to environmental conditions, as well as the relative stability and other emergent properties of the polymer.

In discussing the potential abundance of a prebiotic compound, it is useful to consider the concept of “synthetic depth”. For multi-step reactions in which each reaction is of variable efficiency, the overall yield of any given product may drop quickly. A single low-yielding step can make downstream products extremely scarce. For example, low ammonia concentrations or low ambient pH limit amino acid synthesis via the Strecker mechanism^62^.

Though the conditions which allow for the Strecker synthesis of ɑ-amino acids have been explored extensively (see for example reference^63^), the abiotic synthesis and abundance of some of the monomers described here (e.g., the morpholinones MO and MM) have received little or no attention, though in some cases their prebiotic synthesis may actually be more efficient than that of more conventional biomonomers. For example, ɑHAs are well known to be cosmically abundant^64,65^ and derivable from laboratory simulations^66^, and may in both cases be derived by the cyanohydrin mechanism^3^. 2OX and AEG are products of prebiotic simulation experiments, namely Miller-Urey (MU) type spark discharge experiments and HCN polymerization experiments containing HCHO^46,48^. In fact, the plausibly prebiotic reaction of ethylamine diamine, HCHO and HCN produces AEG via a suggested concerted mechanism which allows it to proceed in high yield (33%) under extremely dilute conditions (10^–6^ M^46^). Cognate prebiotic reactions substituting ethanolamine derivatives or ethylene glycol for ethylene diamine should also yield morpholinones or dioxanones (e.g., DO), respectively.

CM has been detected in the Murchison meteorite^67^, along with various ɑ and Ω-amino acids capable of forming 5- and 6-membered rings^68^ that have also been detected in a variety of prebiotic simulation experiments including HCN polymerizations^4^, MU experiments^12,69^ and Titan tholin simulations^70^. Though to our knowledge they have not been searched for, the cognate lactones or hydroxy acids of such lactams should be accessible by cognate reactions.

After considering the plausibility of synthesis, monomer oligomerizability and stability to the oligomerization conditions are important constraints on the potential relevance of the monomers studied, and their stabilities vary markedly. Some compounds oligomerize readily at relatively low temperature (60 °C, e.g., DO, GA/GD, LA/LD, VN and MO, Fig. 2A–C,F,J) while others do not detectably yield regular oligomers (e.g., CM and TB, Fig. 2E,I). Some degrade markedly at higher temperatures (e.g., 2OX and 1MOX, Fig. SI1G,K), while others are fairly resistant (e.g., GA/GD, VN, Fig. SI1C,F).

The oligomerization of some cyclic monomers is enhanced by the presence of simple amines such as Gly (e.g., DO and VN, Fig. SI5A,F). This can both lead to the synthesis of new mixed oligomers, and increase the oligomerization yield overall.

The diversity of behaviors reported here highlight the complexities of prebiotic chemistry. Even the relatively simple oligomerization of amino acids to the range of lengths (~ 20mers or larger) reported here requires fairly high temperatures (often reported in the literature as ~ 120 °C or greater) or that concentrated reactants be exposed to heat for only short periods of time (see^23,26,31,71^). This still often yields, in addition to oligopeptides, a significant amount of non-peptidic material^71^ or other decomposition products^27,72^, though in the presence of ɑHAs and under specific pH regimes, Gly oligomerization can be easily accomplished without the need for high temperature^54^.

The temperature at which life began remains unknown, and what constitutes “low” and “high” temperatures is relative. It may be that relatively unstable compounds were essential for the origins of life at lower temperature, or even that the origins of life must occur at low temperatures because of the necessity of the involvement of relatively unstable compounds. To put it another way, the monomers need only be stable in the context they operate. For example, the biological polymers that constitute the bulk of modern living cell mass are poorly preserved in terrestrial environments even over relatively short geological timescales^73^. It is thus possible that a wide variety of potentially reactive prebiotically available compounds (e.g., ring monomers in this study and αHAs^45^) that could contribute to protopolymer formation could be challenging to find in meteorites or laboratory simulations of geochemistry.

However, the formation of nano- and microscale aggregates from complex copolymers has been noted in prebiotic contexts repeatedly^27, 58, 74–76^, thus it seems likely these are a general phenomenon to be expected in primordial environmental organic chemistry.

Finally, the “monomers” studied here were investigated due to their known or inferred ease of abiotic synthesis. Although we explored considerable molecular diversity here, there are numerous structural modifications of the compounds in this study that should be similarly polymerizable and may have additional interesting prebiotically relevant properties. The elongation mechanisms discussed here have already been shown to be useful for oligomerizing nucleoside cyclic phosphates^40,41^, and should be applicable to nucleoside analogues as well, of which it seems there could have been a very large prebiotic variety^46,77–80^.

## Conclusions

The abiological synthesis of oligomers, be they proteins or oligoribonucleotides, has long been assumed to be an important step in the origins of life^3^. ɑ-amino acids and activated and unactivated nucleotides can oligomerize when dry-heated or heated at elevated temperature in concentrated solution (e.g.,^23,27,29,31^) but this oligomerization may be inefficient since they are often unstable to prolonged heating at elevated temperatures and significant amounts of starting material are destroyed^24,72,81,82^. We show here that there are a wide variety of alternative, plausibly prebiotic and more reactive monomers that oligomerize without chemical activation at lower temperatures than amino acids do. Some of these oligomers also spontaneously form higher order structures as shown here (Figs. 7, SI6 and SI7) and elsewhere^58^ which may be useful for protocellular organization^58^, though their catalytic capabilities remain underexplored^83^.

The balance between monomer reactivity and polymer stability is related to discussions that the origins of life may represent a transition between thermodynamic and kinetic control^84,85^. The results presented here point to there being heterogeneous polymer systems, not just those studied here, which were easily prebiotically synthesized and could have scaffolded the more precise and kinetically controlled chemistry of modern biology. Due to their ease of synthesis, complex prebiotic *xeno*-polymer systems may have facilitated the exploration of catalytic sequence space before modern polymer systems were able to do so^83^.

## Materials and methods

All chemicals (unless otherwise noted) were purchased from Sigma-Aldrich, Wako or Tokyo Chemical Industry (TCI) (all from Tokyo, Japan) and were of reagent grade or higher and used without further purification. 2-Oxopiperazine (2OX) was purchased from Enamine (Kiev, Ukraine).

All glassware and metalware was heated at 500 °C for 3 h to eliminate potential organic contaminants. Water used for experiments was from a Milli-Q Integral 3 Water Purification system (Merck, Tokyo, Japan), and was of 18.2 M conductivity at 25 °C and contained a maximum of 3 ppb Total Organic Carbon (TOC).

Drying reactions were initially 100 µL in volume with a total monomer concentration of 1 M in water, with the pH unadjusted unless otherwise noted. Reactions of ROP monomers with Gly were conducted in a 1:1 ratio. Drying experiments were conducted in open 13 mm × 100 mm borosilicate test tubes under air. Reactions were held at constant temperature (± 0.1 °C) using Sahara 310 dry heating baths (Rocker Scientific, New Taipei City, Republic of China) and monitored by conventional liquid thermometers. The oligomerization of each monomer was investigated between 60–100 °C by allowing reactions to dry for 24 h, followed by rehydration immediately prior to analysis. Photographs in Figure SI3 were obtained using an iPhone SE (Apple, Cupertino, CA, USA).

### NMR spectroscopy

^1^H NMR spectra were recorded on either a Varian Mercury 400 MHz or Bruker DRX 500 MHz spectrometer (Bruker, Massachusetts, USA). Diffusion Ordered Spectroscopy (DOSY) experiments were performed using a longitudinal encode-decode (LED) pulse sequence with a 5–95% varying gradient strength over 19 points.

2OX was dissolved in buffers adjusted to a known pH (+/− 0.2 pH units) as measured using either pH paper (Sigma-Aldrich, Missouri, USA) or a pH meter (Mettler Toledo, Ohio, USA). Buffers used were 0.5 M pH 5 sodium acetate, pH 7 sodium phosphate or pH 9 sodium carbonate. Buffers were prepared at the appropriate pH in water, then exchanged 3 × in 99.8% D_2_O under vacuum. Reactions were held at constant temperature in a thermostatted oven for periods of up to 6 months.

NMR measurements of 2OX were recorded starting from concentrations of 2, 20 or 200 mM using ~ 0.5 mL volumes in Wilmad NMR tubes (Wilmad Lab Glass, New Jersey, USA) capped under air. Samples were measured periodically, and the kinetics measured by comparison of proton integrations. Results reported in Figure SI4 were conducted at 25° C or 40° C as noted.

### Electrospray ionization mass spectrometry (ESI–MS)

After rehydration, samples were diluted 1000 × with water prior to MS analysis. Electrospray Ionization Quadrupole Time-of-Flight Mass Spectrometry (ESI-QToF-MS) analysis was carried out by direct infusion at a flow rate of 0.4 ml min^−1^ using a Waters Xevo G2-XS QToF-MS (Waters, Tokyo, Japan) operated in positive or negative mode. Source settings were as follows: positive and negative-mode: ion source temperature 150 °C, desolvation gas temperature 550 °C, cone voltage 20 V, cone gas flow rate 50 l h^−1^ and desolvation gas flow rate 1000 l h^−1^. The capillary voltage was 1.2 kV in negative mode and 1.0 kV in positive mode. For MS/MS experiments, the data for which are presented for select oligomer systems, collision-induced dissociation energies were set to 6 eV and the isolation window was ~ 3 Da. A water blank was injected every five injections to guard against signal carry-over between injections. Fragmentation spectra were collected generally with a ~ 3 Da isolation window. MS data analysis was conducted according to the methods described in Chandru et al.^51^ Products corresponding to expected species within 5 ppm mass accuracy were assigned. Masslynx (Waters, Massachusetts, USA), mMass (Prague, Czech Republic)^86–88^ and Adobe Illustrator (California, USA) were used for basic data processing and figure preparation, respectively.

### Light microscopy

Micrographs were obtained either using an Olympus (Tokyo, Japan) IX73 inverted fluorescent microscope on a 40 × 0.60 air Ph2 LUCPlanFL objective or a 100X 1.30 Oil Ph3 UPlanFL N objective (Fig. 7), or a Leica (Wetzlar, Germany) DM5500 B automated upright fluorescence microscope with an HCX PL FLUOTAR 100X/1.30 Oil Ph 3 or a HC PL FLUOTAR 40x/0.80 PH2 air objective (Fig. SI7). Briefly, 100 µL solutions of 500 mM glycolide and 500 mM glycine were heated at 80 °C for 48 h in 13 mm × 100 mm borosilicate glass culture tubes in a Sahara 310 dry heating bath. Afterwards, the resulting tar-like substance was rehydrated in 100 µL of water. Samples were prepared by first punching a hole into double-sided tape (strong type, Naisutakku, Nichiban KK, Tokyo, Japan), and then depositing the double-sided tape onto a 76 mm × 26 mm × 1 mm slide glass (Lauda-Königshofen, Germany). 3.5 µL of the rehydrated sample was deposited in this hole, followed by covering with a cover slip (18 × 18 mm, No. 1 0.12–0.17 mm, Matsunami Glass Ind., Ltd., Osaka-fu, Japan). Images were acquired by Metamorph (Molecular Devices, Tokyo, Japan) or Leica LAS X software, and then analyzed by FIJI (Fiji is Just ImageJ, https://fiji.sc).

### Droplet size analysis

For image analysis using FIJI, "Huang" thresholding was used, in addition to the “Analyze Particles” function with no size filter and a circularity filter of Circularity = 0.70–1.00. We also further excluded any droplets that were on the edge of the image, droplets which were smaller than 1 μm^2^, and droplets which were not reasonably circular (as defined by the “Circularity” function; this included droplets which overlapped after thresholding due to close distance to each other). All droplets were assumed to be spheres, and thus the diameter of each particle was back-calculated from its computed area.

## Supplementary information

Supplementary file1

## Data Availability

All data are available from the corresponding author upon request.
