# Supplementary material for: Prebiotic oligomerization and self-assembly of structurally diverse xenobiological monomers
Source: Sci Rep. 2020 Oct 16;10:17560. doi: 10.1038/s41598-020-74223-5 (PMC7567815; doi:10.1038/s41598-020-74223-5)
Supplement: Supplementary file 1 — Supplementary file1 [file 41598_2020_74223_MOESM1_ESM.pdf]

Supporting Information

Prebiotic Oligomerization and Self-Assembly of Structurally Diverse Xenobiological Monomers

Kuhan Chandru[1,2*], Tony Z. Jia[3,4], Irena Mamajanov[3], Niraja Bapat[3,5], and H. James Cleaves II[3,4,6]

1. Space Science Center (ANGKASA), Institute of Climate Change, Level 3, Research Complex, National University of Malaysia, 43600, UKM Bangi, Selangor, Malaysia
2. Department of Physical Chemistry, University of Chemistry and Technology, Prague, Technicka 5, 16628, Prague 6 – Dejvice, Czech Republic
3. Earth-Life Science Institute, Tokyo Institute of Technology, 2-12-1-IE-1 Ookayama, Meguro-ku, Tokyo 152-8550, Japan
4. Blue Marble Space Institute for Science, 1001 4th Ave, Suite 3201, Seattle, WA 98154, USA
5. Indian Institute of Science Education and Research, Dr. Homi Bhabha Road, Pashan, Pune, 411 008, Maharashtra, India
6. Institute for Advanced Study, 1 Einstein Drive, Princeton, NJ 08540, USA

*Corresponding Author: kuhan@ukm.edu.my

Abbreviations

1,4-Dioxan-2-one (DO); Lactide (LD); Glycolide (GD); ε-Caprolactone (CN); ε-Caprolactam (CM); δ-Valerolactone (VN); 2-Oxopiperazine (2OX); 4-Methylmorpholin-2-one (MM); γ-Thiobutyrolactone (TB); Morpholine-2-one (MO); 1-Methyl-3-oxopiperazine (1MOX); Glycolic acid (GA); Lactic acid (LA) and Glycine (Gly)

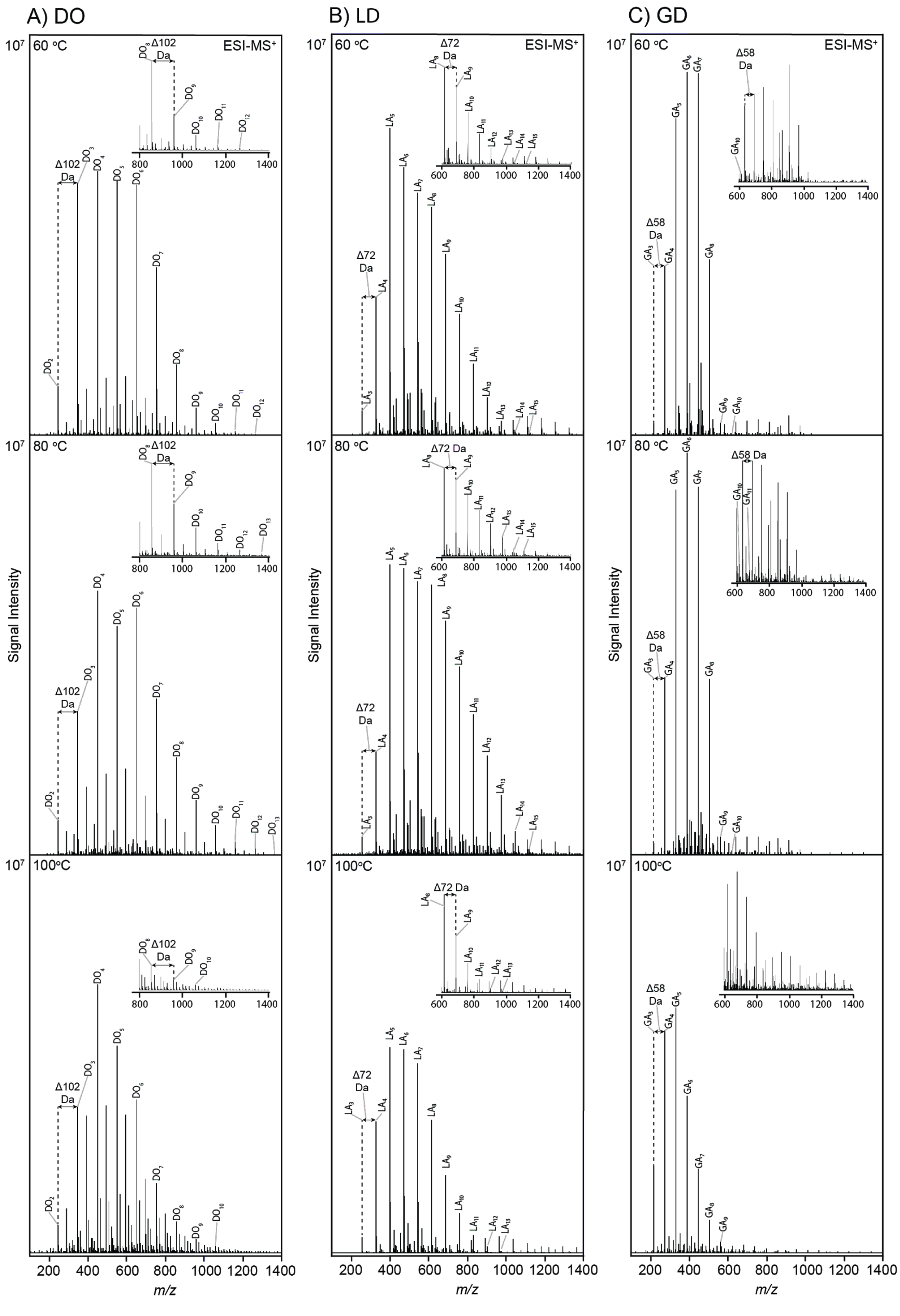

A) DO
B) LD
C) GD
ESI-MS+
60 °C
80 °C
100°C
Δ102 Da
Δ72 Da
Δ58 Da
Signal Intensity
10^7
m/z

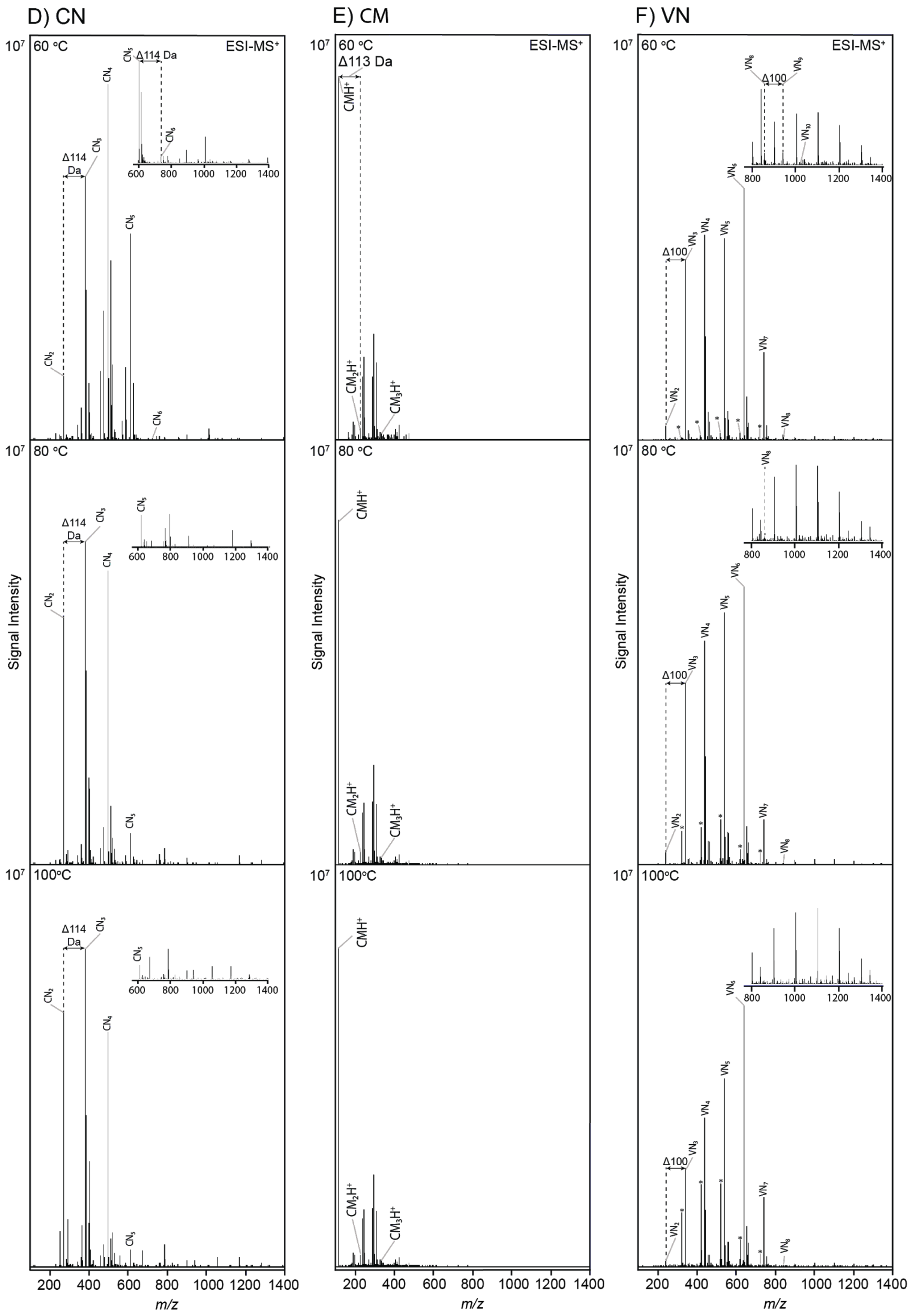

D) CN
E) CM
F) VN
ESI-MS+
60 °C
80 °C
100°C
Δ114 Da
Δ113 Da
Δ100
CMH+
CM2H+
CM3H+
Signal Intensity
m/z

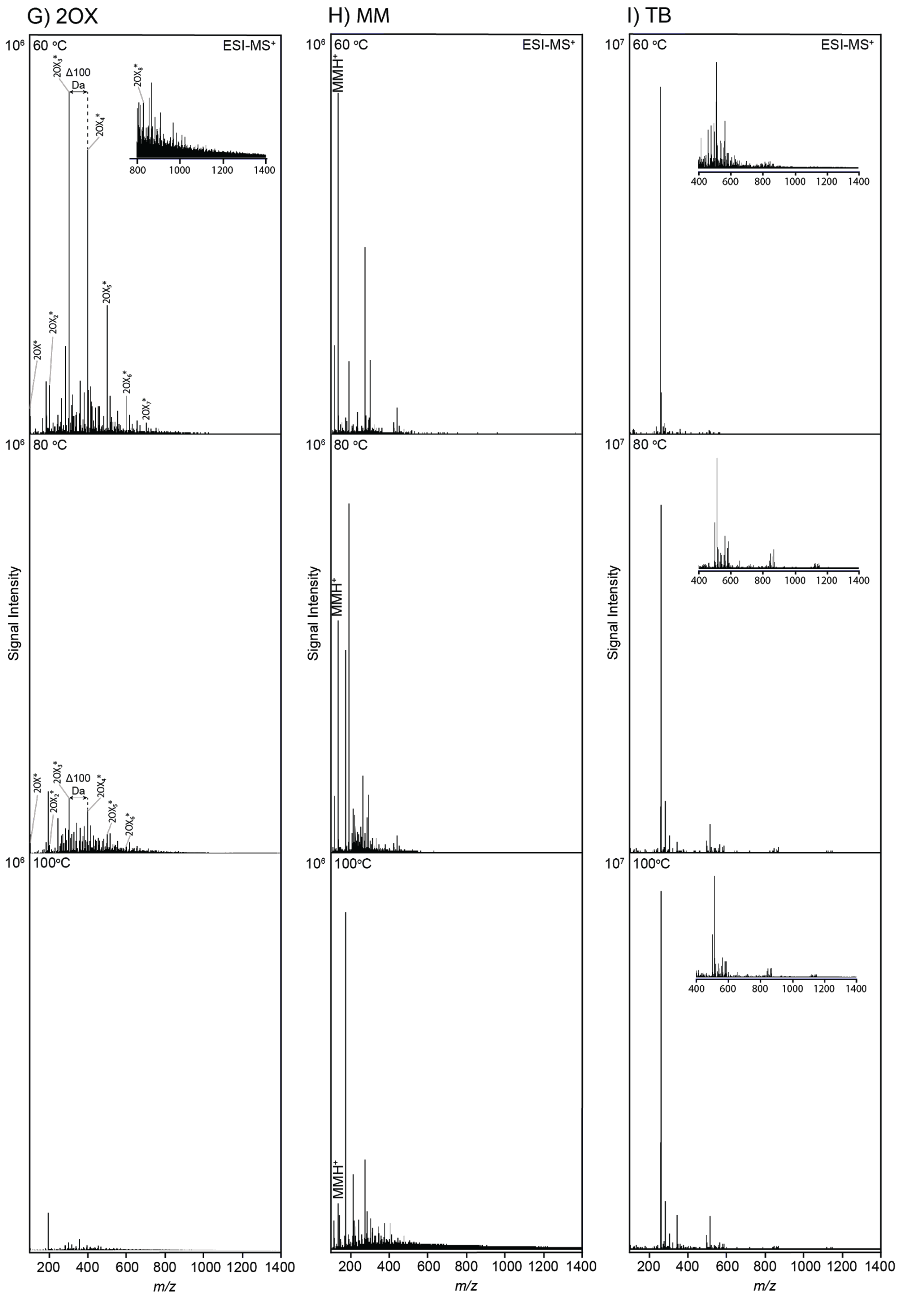
G) 2OX
H) MM
I) TB
60 °C
80 °C
100°C
ESI-MS+
Δ100 Da
MMH+
Signal Intensity
m/z
10^6
10^7
200 400 600 800 1000 1200 1400

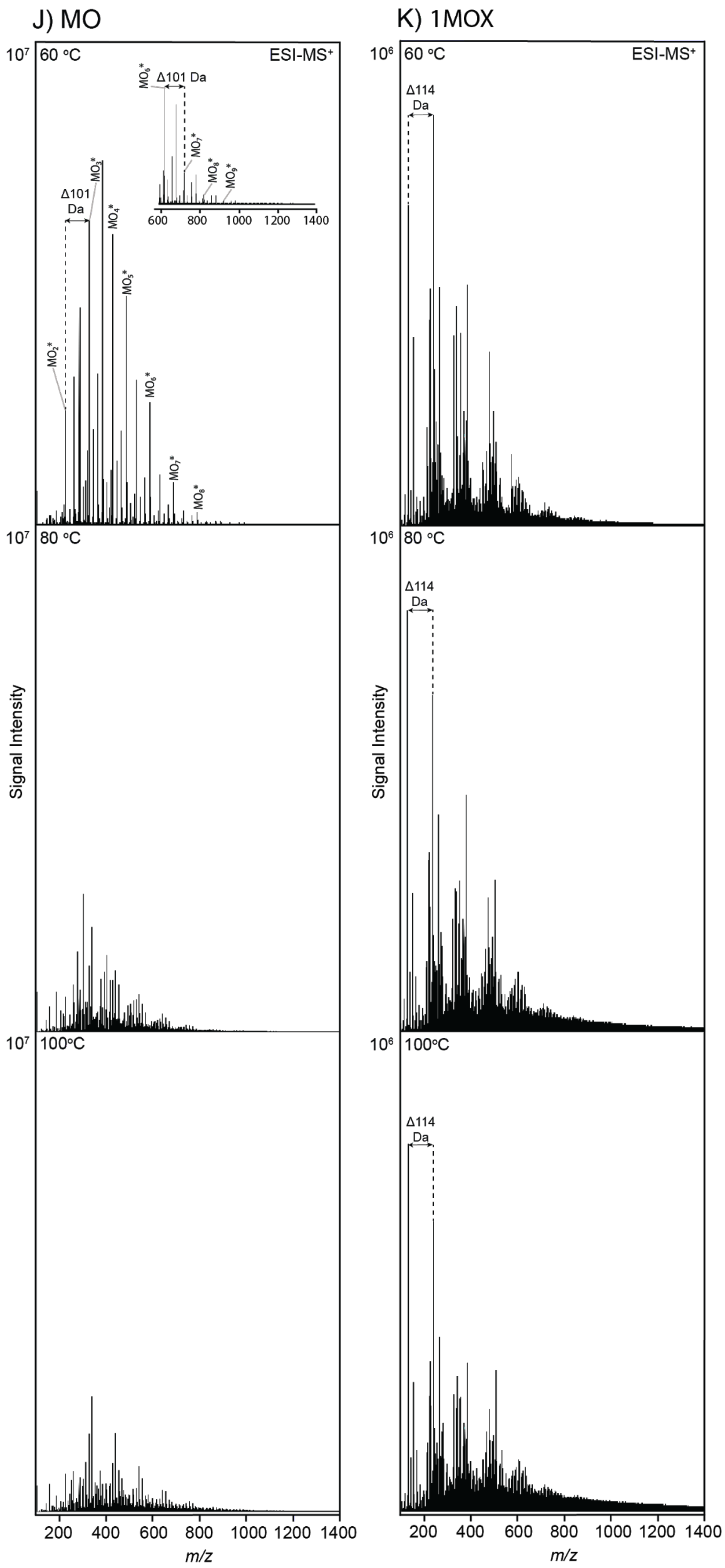

J) MO
K) 1MOX
60 °C
80 °C
100°C
ESI-MS+
10^7
10^6
Δ101 Da
Δ114 Da
MO2*
MO3*
MO4*
MO5*
MO6*
MO7*
MO8*
MO9*
Signal Intensity
m/z
200 400 600 800 1000 1200 1400

Figure SI1. Positive mode Electrospray Ionization Quadrupole Time-of-Flight Mass Spectrometry (ESI-QToF-MS) of oligomers formed at various temperatures as indicated. Labeled peaks were detected as parent mass plus sodium (MNa^+) adducts for all compounds except 2OX for which oligomers were detected as $MH-H_2O^+$ adducts, and CM and MM which were detected as MH^+ adducts. Asterisk-labeled peaks indicate water-loss $MNa-H_2O^+$. Note 3: MK^+ and MNH_4^+ adduct peaks were also detected but not shown here due to space limitations.

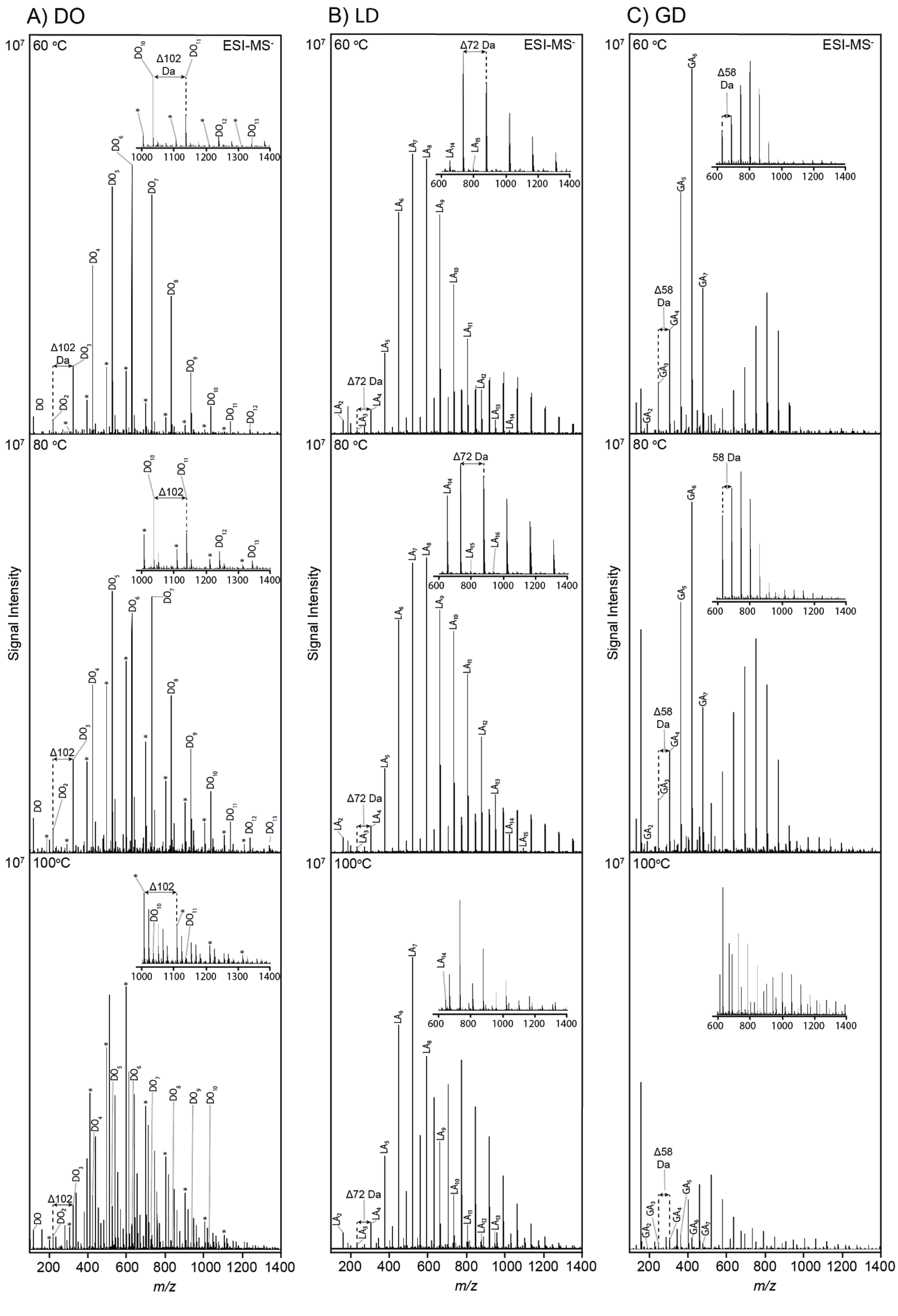
A) DO
B) LD
C) GD
ESI-MS⁻
60 °C
80 °C
100°C
Signal Intensity
m/z
Δ102 Da
Δ72 Da
Δ58 Da
58 Da

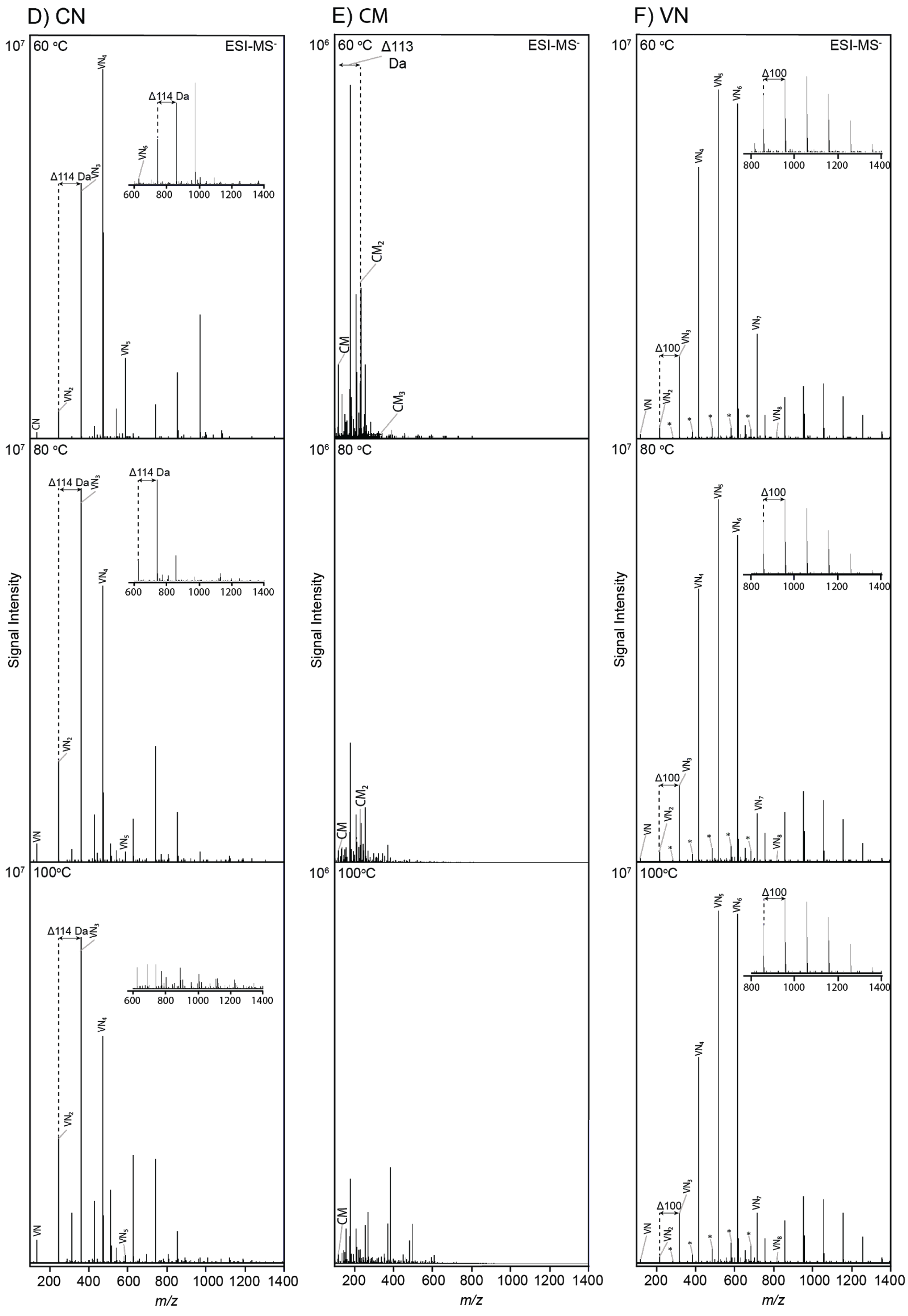

D) CN
E) CM
F) VN
60 °C
80 °C
100°C
ESI-MS⁻
Δ114 Da
Δ113 Da
Δ100
Signal Intensity
m/z

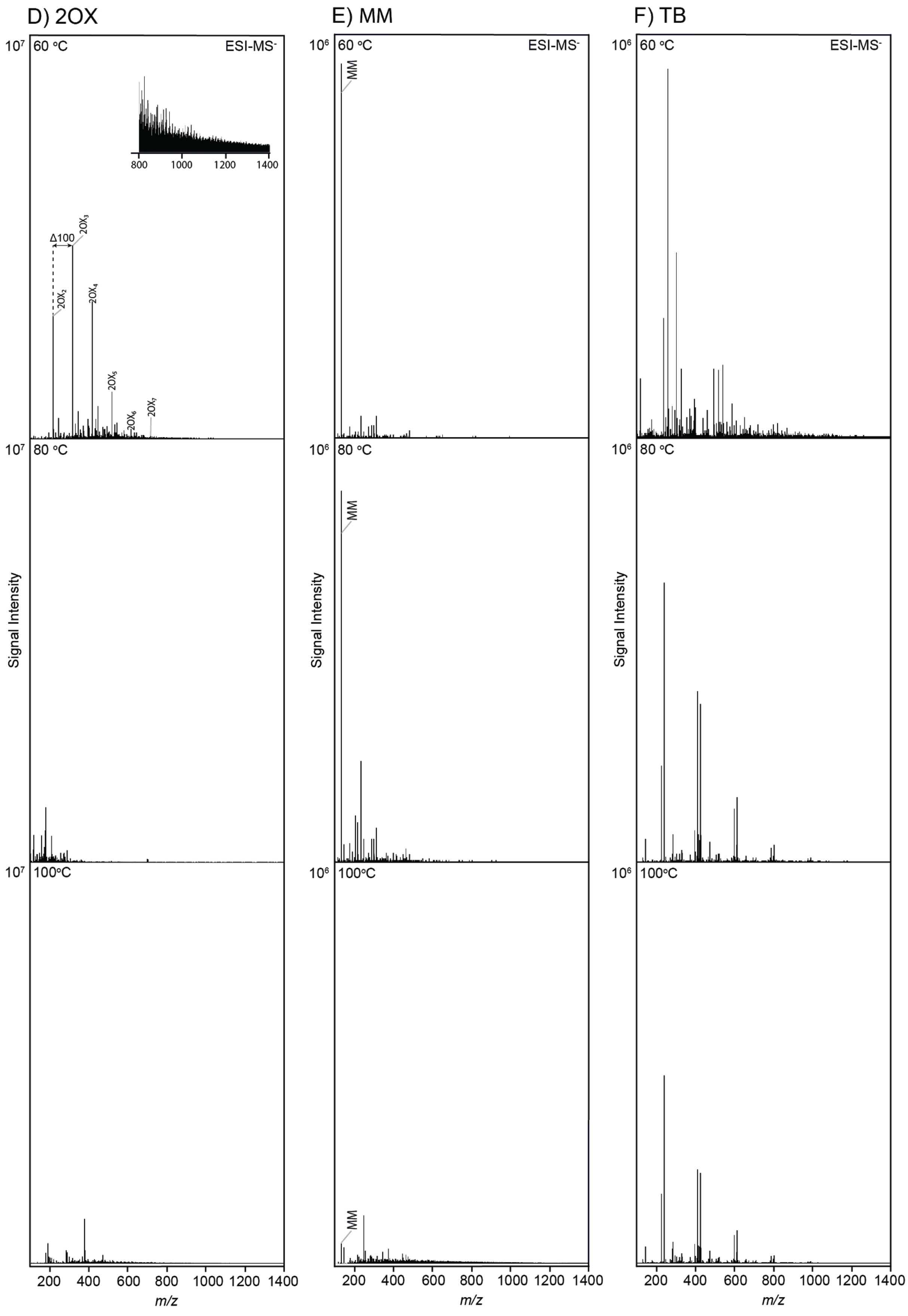

D) 2OX
E) MM
F) TB
60 °C
80 °C
100°C
ESI-MS-
10^7
10^6
Signal Intensity
m/z
Δ100
2OX2
2OX3
2OX4
2OX5
2OX6
2OX7
MM
200 400 600 800 1000 1200 1400
800 1000 1200 1400

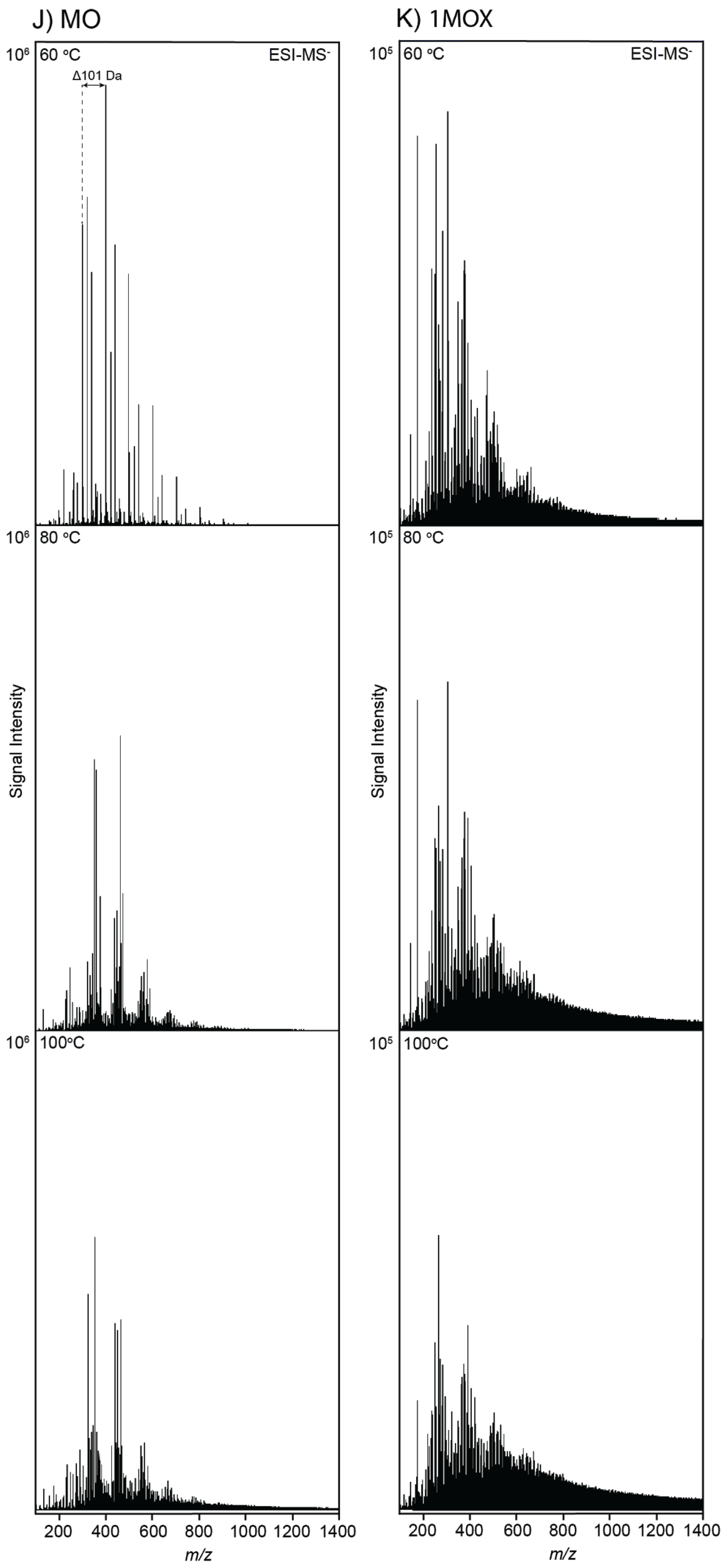
J) MO
K) 1MOX
10⁶
10⁵
60 °C
80 °C
100°C
ESI-MS⁻
Δ101 Da
Signal Intensity
200
400
600
800
1000
1200
1400
m/z

Figure SI2. Negative mode ESI-QToF-MS of oligomers formed at various temperatures. Labeled peaks are MH^- adducts.

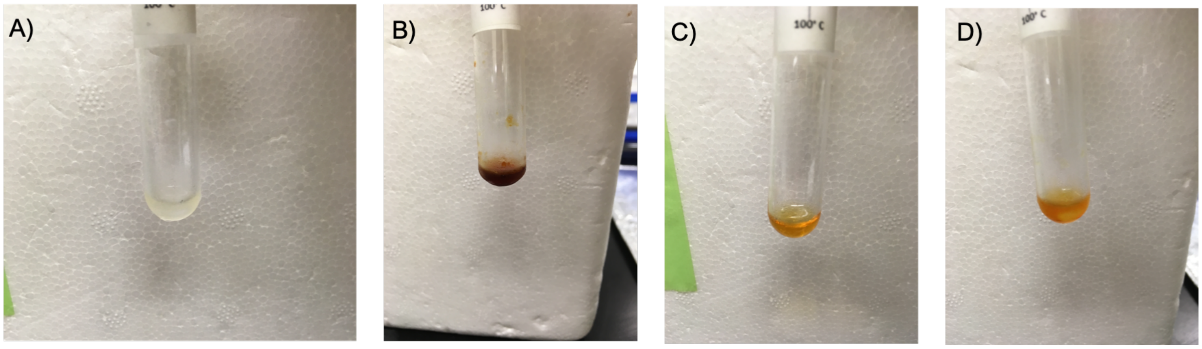

Figure SI3: Examples of the browning observed in samples after heating at 100° C. A) 1MO, B) 2OX, C) MM and D) 1MOX.

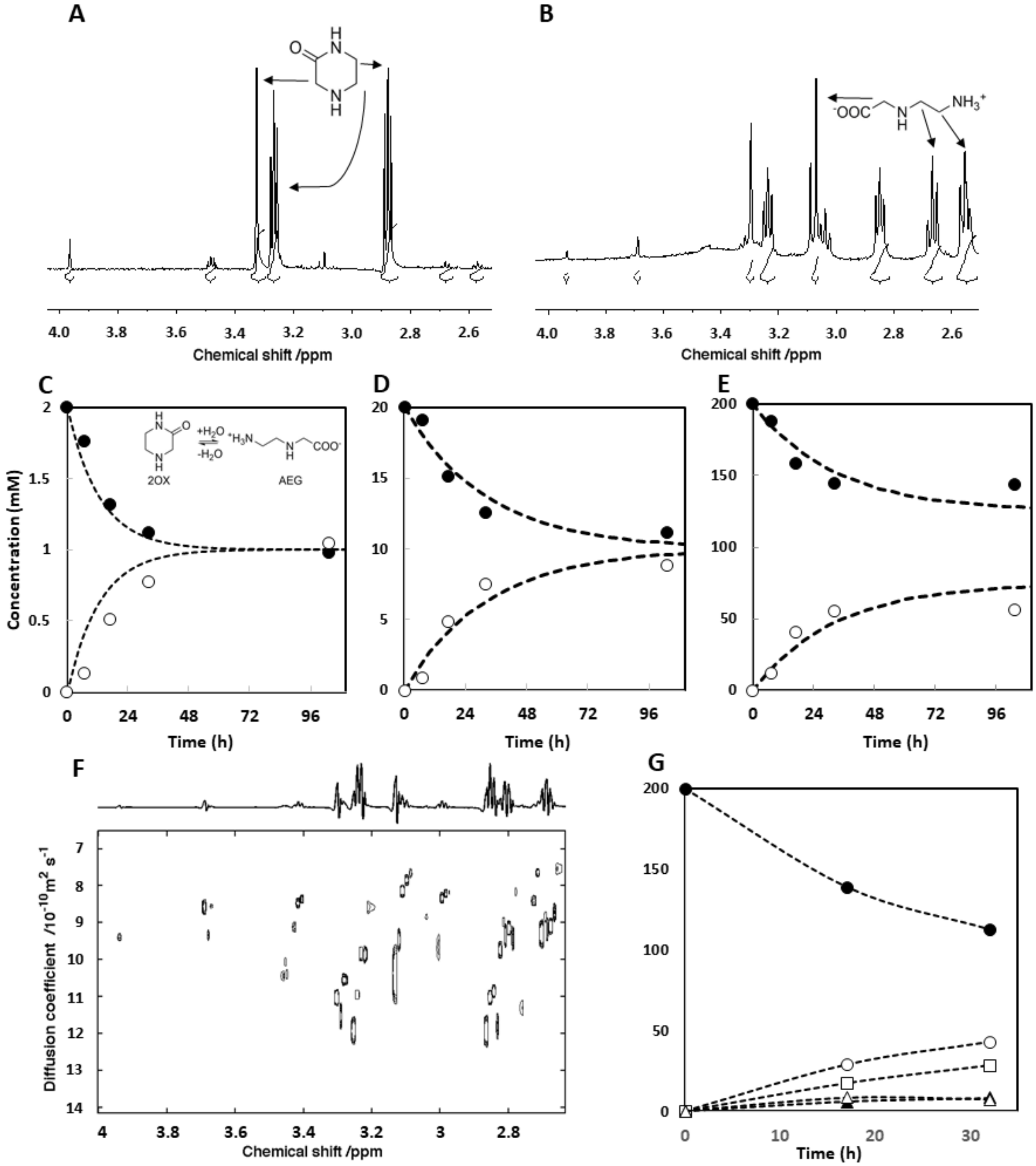

Figure SI4. ^{1}H NMR monitoring of the ring-opening reaction of 2OX at pH 9 at different concentrations. Proton shift assignments at 0 (A) and 96 hours (B) at 25° C. Conversion of 2OX to AEG as a function of time and initial 2OX concentration (2 mM (C), 20 mM (D) and 200 mM (E)) at 25° C and pH 9. Dotted line curve fits are merely intended as guides for the eye. DOSY spectrum (F) of a reaction of 200 mM 2OX at pH 9 and 40° C (G), showing the formation of oligomers in solution. In all plots black circles are 2OX and white circles are AEG. In (G), squares are peaks assigned as dimers, white triangles are peaks assigned as trimers and black triangles, which largely superpose with assigned trimers in this plot, are peaks assigned as tetramers.

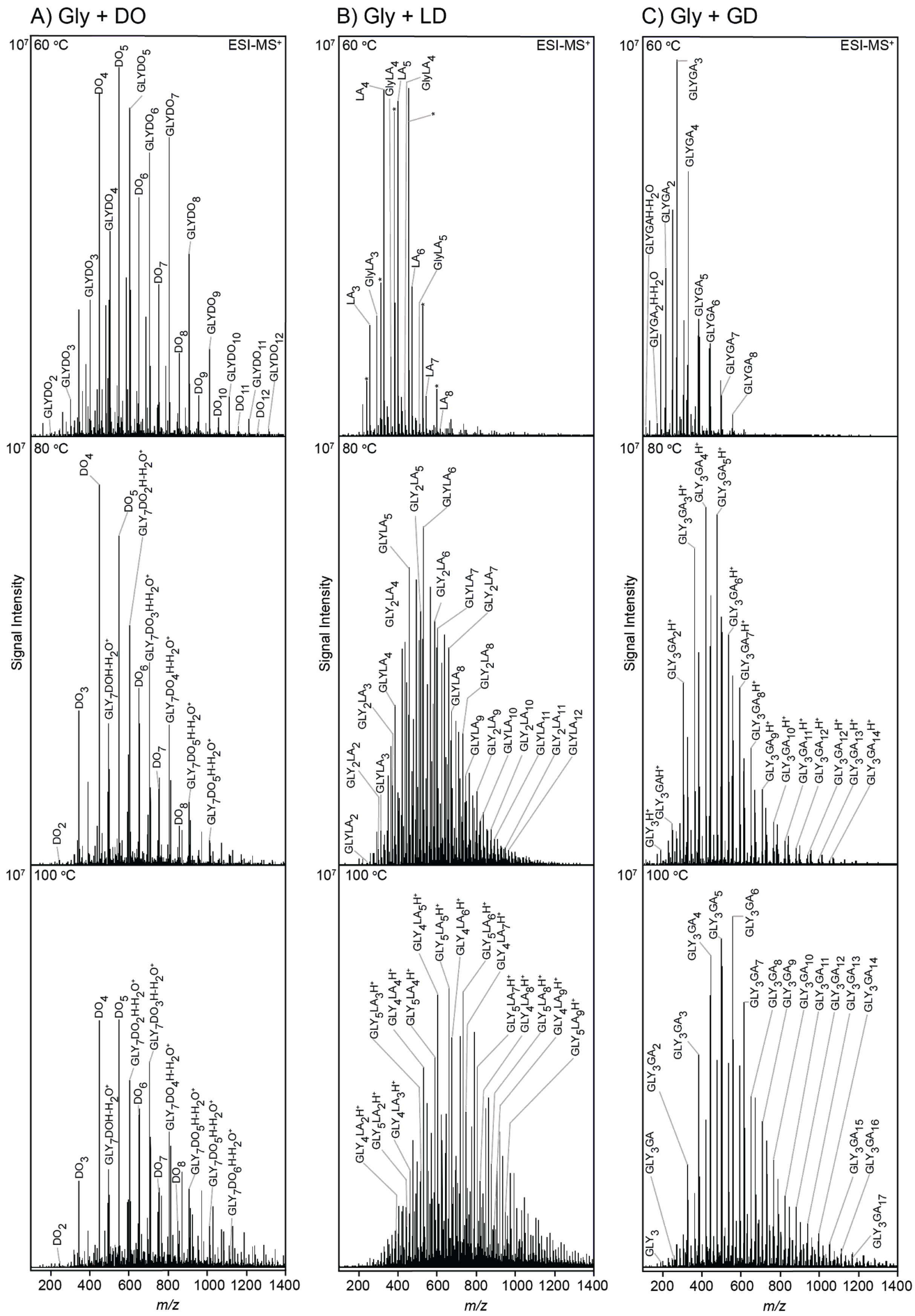

A) Gly + DO
B) Gly + LD
C) Gly + GD
ESI-MS+
60 °C
80 °C
100 °C
10^7
Signal Intensity
m/z
200 400 600 800 1000 1200 1400
GLYDO2
GLYDO3
GLYDO4
GLYDO5
GLYDO6
GLYDO7
GLYDO8
GLYDO9
GLYDO10
GLYDO11
GLYDO12
DO2
DO3
DO4
DO5
DO6
DO7
DO8
DO9
DO10
DO11
DO12
GLY7DOH-H2O+
GLY7DO2H-H2O+
GLY7DO3H-H2O+
GLY7DO4H-H2O+
GLY7DO5H-H2O+
GLY7DO6H-H2O+
LA3
LA4
LA5
LA6
LA7
LA8
GlyLA3
GlyLA4
GlyLA5
GLYLA2
GLYLA3
GLYLA4
GLYLA5
GLYLA6
GLYLA7
GLYLA8
GLYLA9
GLYLA10
GLYLA11
GLYLA12
GLY2LA2
GLY2LA3
GLY2LA4
GLY2LA5
GLY2LA6
GLY2LA7
GLY2LA8
GLY2LA9
GLY2LA10
GLY2LA11
GLY4LA2H+
GLY4LA3H+
GLY4LA4H+
GLY4LA5H+
GLY4LA6H+
GLY4LA7H+
GLY4LA8H+
GLY4LA9H+
GLY5LA2H+
GLY5LA3H+
GLY5LA4H+
GLY5LA5H+
GLY5LA6H+
GLY5LA7H+
GLY5LA8H+
GLY5LA9H+
GLYGAH-H2O
GLYGA2H-H2O
GLYGA2
GLYGA3
GLYGA4
GLYGA5
GLYGA6
GLYGA7
GLYGA8
GLY3H+
GLY3GAH+
GLY3GA2H+
GLY3GA3H+
GLY3GA4H+
GLY3GA5H+
GLY3GA6H+
GLY3GA7H+
GLY3GA8H+
GLY3GA9H+
GLY3GA10H+
GLY3GA11H+
GLY3GA12H+
GLY3GA13H+
GLY3GA14H+
GLY3
GLY3GA
GLY3GA2
GLY3GA3
GLY3GA4
GLY3GA5
GLY3GA6
GLY3GA7
GLY3GA8
GLY3GA9
GLY3GA10
GLY3GA11
GLY3GA12
GLY3GA13
GLY3GA14
GLY3GA15
GLY3GA16
GLY3GA17

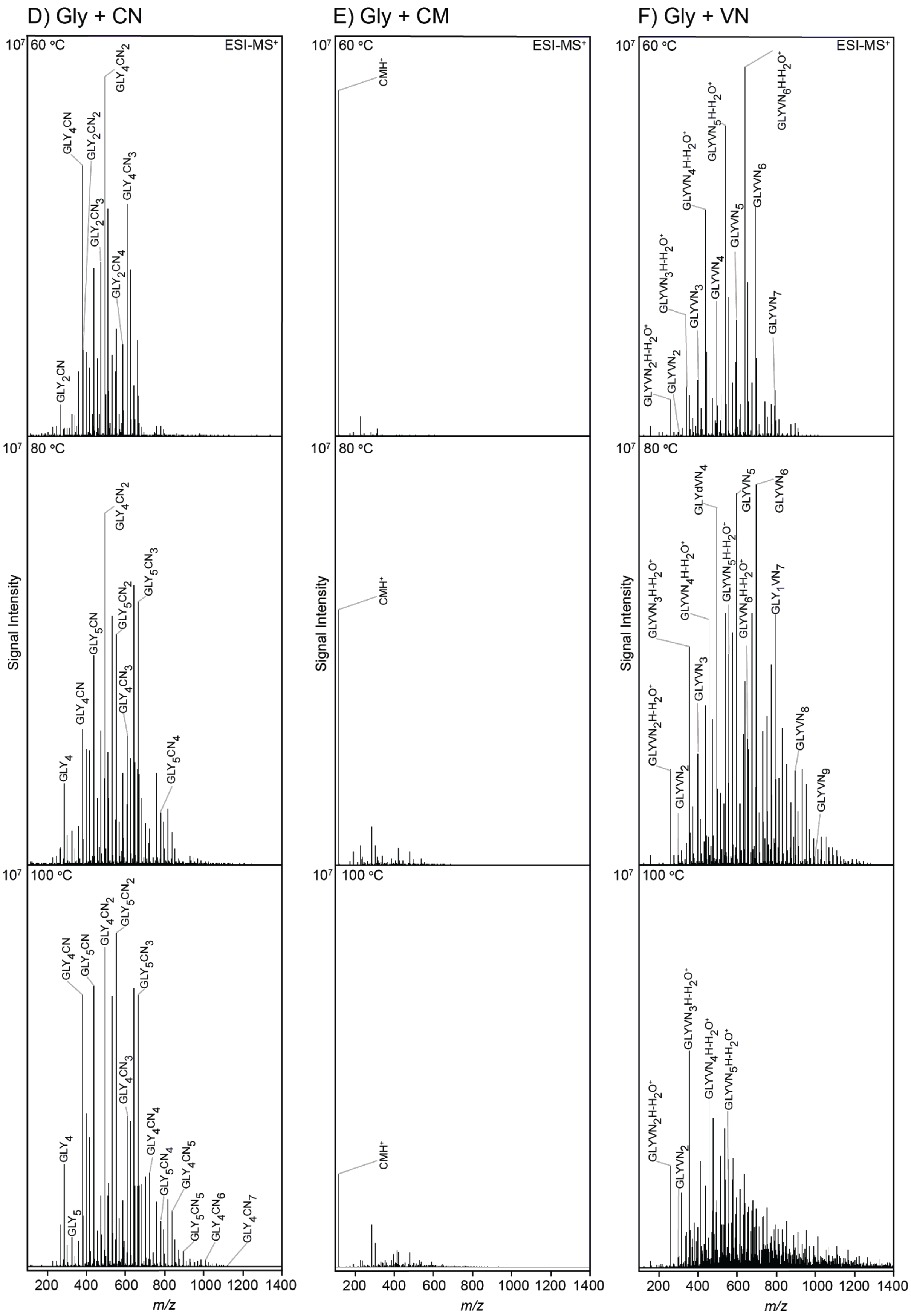
D) Gly + CN
E) Gly + CM
F) Gly + VN
ESI-MS+
60 °C
80 °C
100 °C
10^7
Signal Intensity
m/z
200 400 600 800 1000 1200 1400
GLY2CN
GLY4CN
GLY2CN2
GLY4CN2
GLY2CN3
GLY4CN3
GLY2CN4
GLY4
GLY5CN
GLY5CN2
GLY5CN3
GLY5CN4
GLY5
GLY4CN4
GLY4CN5
GLY5CN5
GLY4CN6
GLY4CN7
CMH+
GLYVN2H-H2O+
GLYVN2
GLYVN3H-H2O+
GLYVN3
GLYVN4H-H2O+
GLYVN4
GLYVN5H-H2O+
GLYVN5
GLYVN6H-H2O+
GLYVN6
GLYVN7
GLYdVN4
GLY1VN7
GLYVN8
GLYVN9

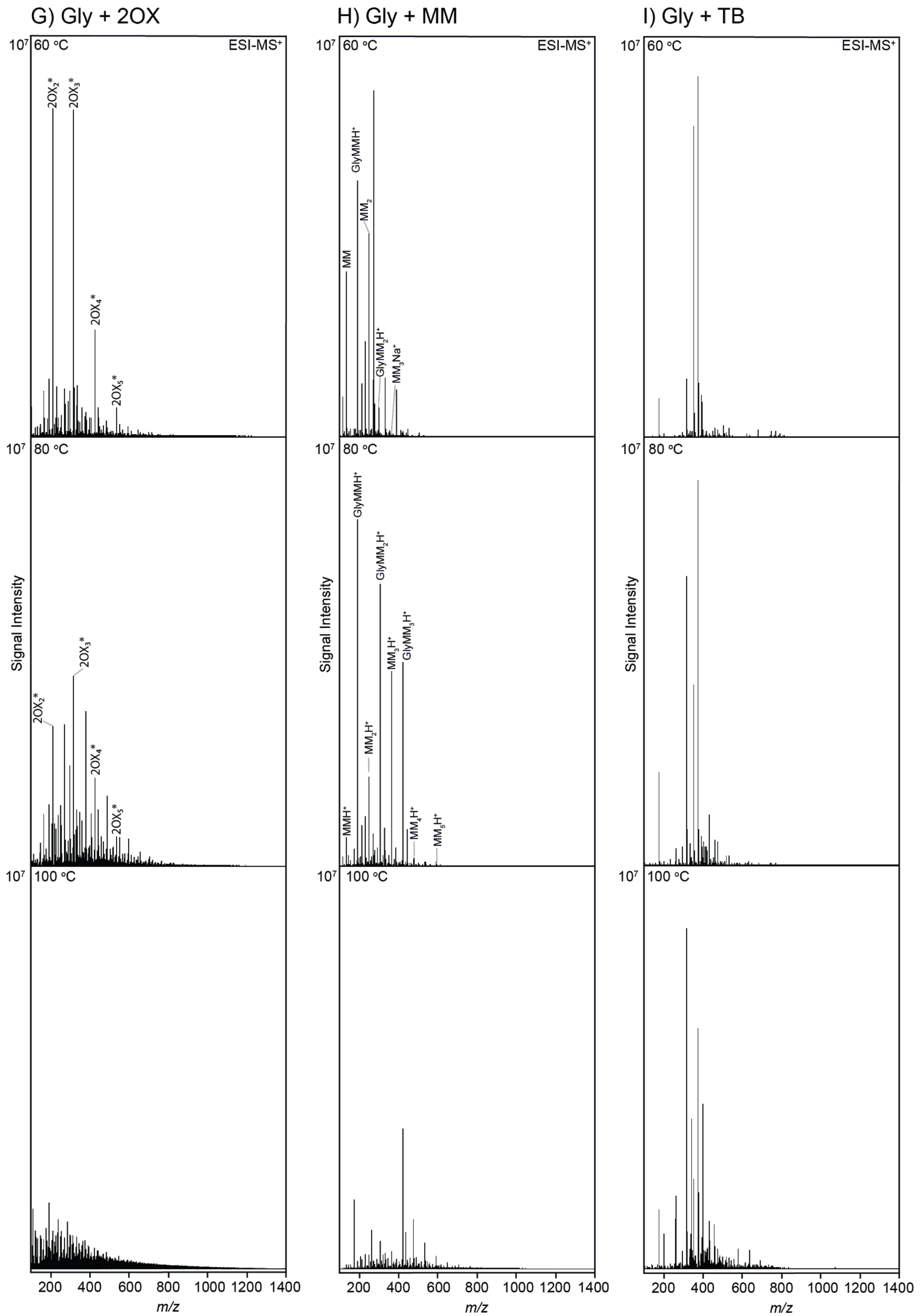

G) Gly + 2OX
H) Gly + MM
I) Gly + TB
60 °C
80 °C
100 °C
ESI-MS+
10^7
Signal Intensity
m/z
2OX2*
2OX3*
2OX4*
2OX5*
MM
GlyMMH+
MM2
GlyMM2H+
MM3Na+
MMH+
MM2H+
MM3H+
GlyMM3H+
MM4H+
MM5H+
200
400
600
800
1000
1200
1400

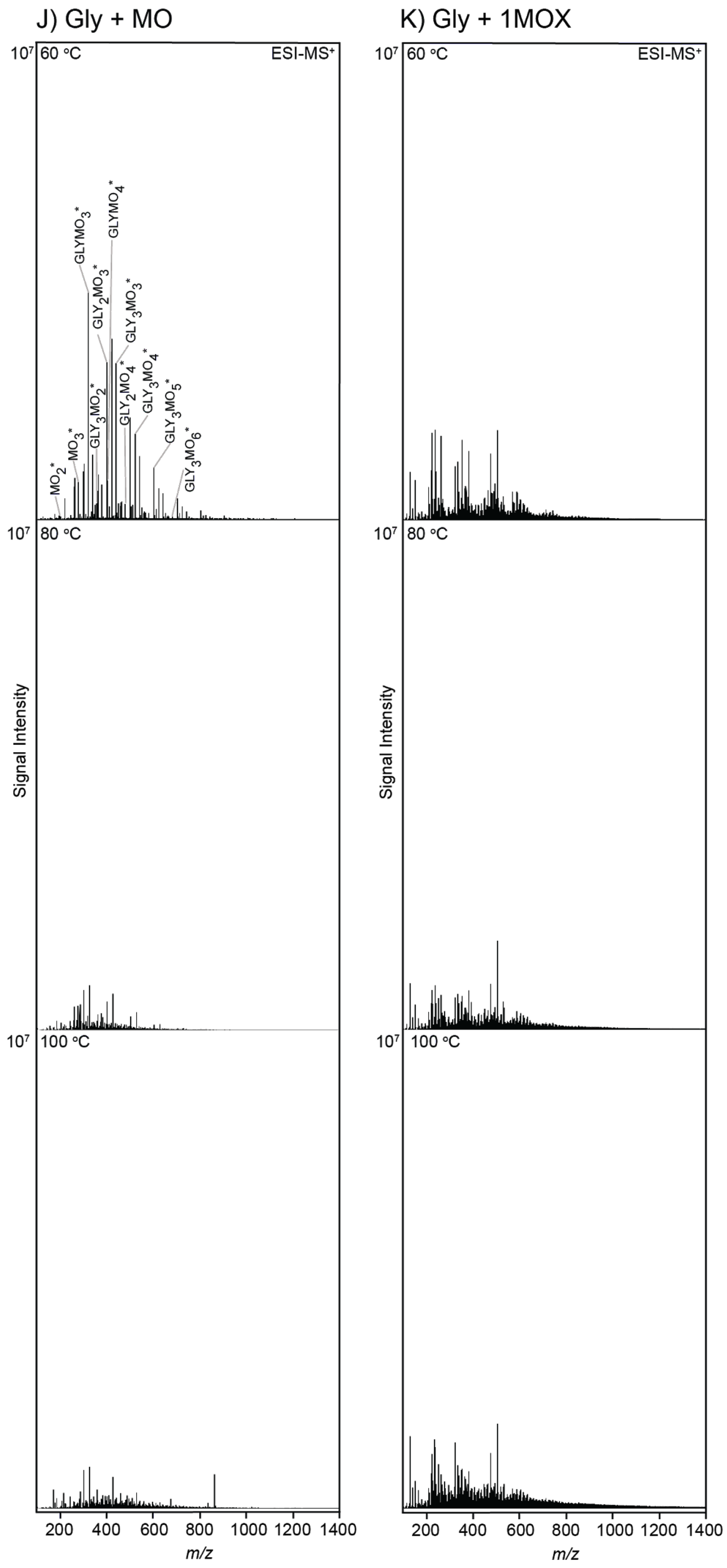

J) Gly + MO
K) Gly + 1MOX
10^7
60 °C
80 °C
100 °C
ESI-MS+
Signal Intensity
MO2*
MO3*
GLY3MO2*
GLYMO3*
GLY2MO3*
GLYMO4*
GLY3MO3*
GLY2MO4*
GLY3MO4*
GLY3MO5*
GLY3MO6*
200 400 600 800 1000 1200 1400
m/z

Figure SI5: Positive mode ESI-QToF-MS of cyclic monomers reacted with Gly at the indicated temperatures. Labeled peaks were detected as parent mass plus sodium (MNa^+) adducts for all compounds except for some spectra. Asterisk-labeled peaks indicate sodium adducts of peaks also suggestive of water-loss ($MNa\text{-}H_2O^+$). Only major peak series of LD and GD are shown in (B) and (C).

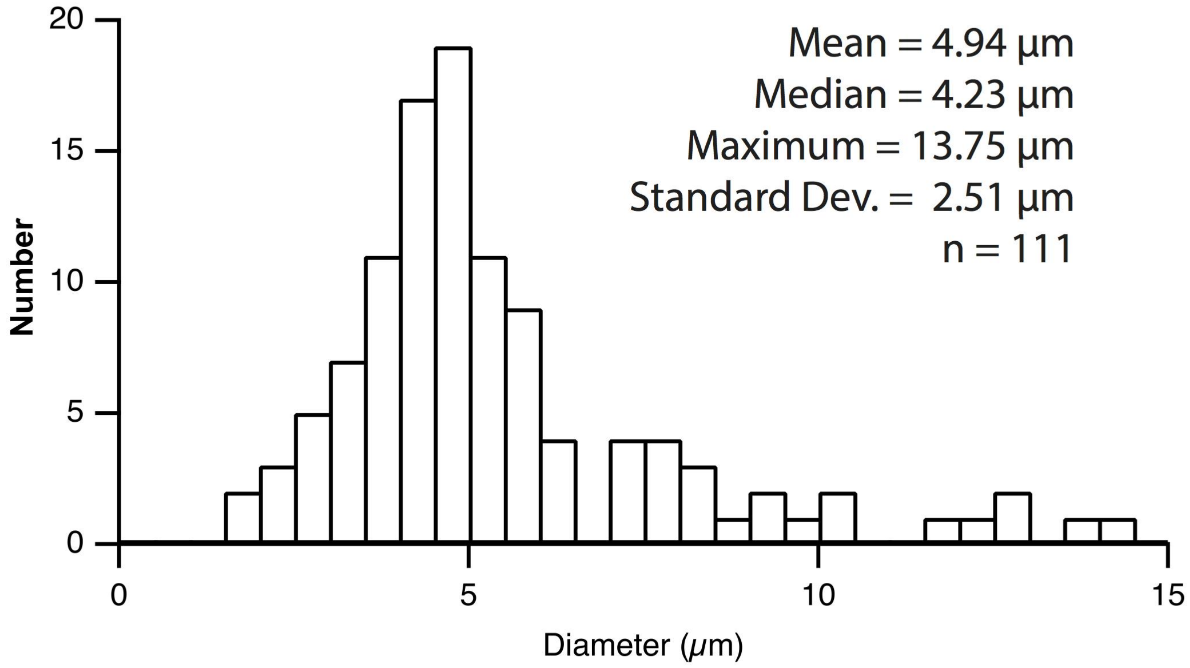

Figure SI6. Histogram showing size distributions of the spherical particles (diameter in µm) from Fig. 7. The mean, median, maximum, and standard deviation of particle diameters and the number of particles (n) detected are also shown.

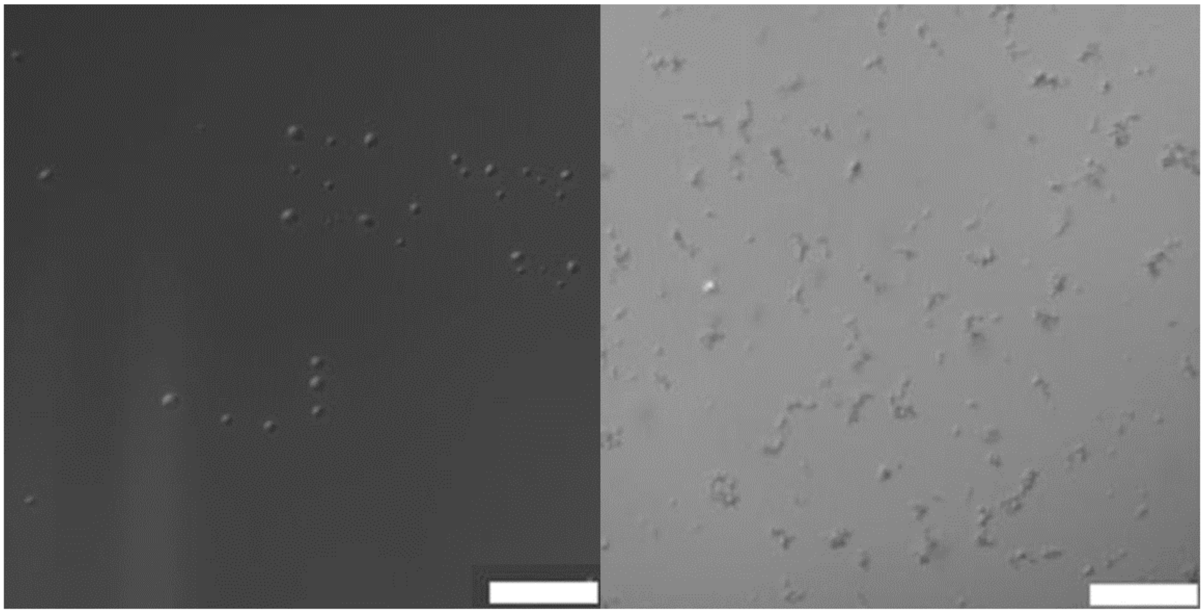

Figure SI7. Micrograph of the structures obtained after rehydration of the drying reaction of GD and Gly at 80° C. Scale bar is 100 µm. In the same sample, both droplets (left) and rod-like aggregates (right) were observed (albeit in different focal planes).
